# Targeting esophageal carcinoma: molecular mechanisms and clinical studies

**DOI:** 10.1002/mco2.782

**Published:** 2024-10-15

**Authors:** Wenjing Wang, Lisha Ye, Huihui Li, Weimin Mao, Xiaoling Xu

**Affiliations:** ^1^ Department of Medical Thoracic Oncology Zhejiang Cancer Hospital, Hangzhou Institute of Medicine (HIM), Chinese Academy of Sciences Hangzhou Zhejiang China; ^2^ Postgraduate Training Base Alliance Wenzhou Medical University Wenzhou Zhejiang China; ^3^ The Cancer Hospital of the University of Chinese Academy of Sciences, Institute of Basic Medicine and Cancer (IBMC) Chinese Academy of Sciences Hangzhou Zhejiang China; ^4^ Department of Radiation Oncology Shanghai Pulmonary Hospital, Tongji University School of Medicine Shanghai China

**Keywords:** combination therapies, esophageal cancer, molecular pathogenesis, resistance mechanisms, target therapy

## Abstract

Esophageal cancer (EC) is identified as a predominant health threat worldwide, with its highest incidence and mortality rates reported in China. The complex molecular mechanisms underlying EC, coupled with the differential incidence of esophageal squamous cell carcinoma (ESCC) and esophageal adenocarcinoma (EAC) across various regions, highlight the necessity for in‐depth research targeting molecular pathogenesis and innovative treatment strategies. Despite recent progress in targeted therapy and immunotherapy, challenges such as drug resistance and the lack of effective biomarkers for patient selection persist, impeding the optimization of therapeutic outcomes. Our review delves into the molecular pathology of EC, emphasizing genetic and epigenetic alterations, aberrant signaling pathways, tumor microenvironment factors, and the mechanisms of metastasis and immune evasion. We further scrutinize the current landscape of targeted therapies, including the roles of EGFR, HER2, and VEGFR, alongside the transformative impact of ICIs. The discussion extends to evaluating combination therapies, spotlighting the synergy between targeted and immune‐mediated treatments, and introduces the burgeoning domain of antibody–drug conjugates, bispecific antibodies, and multitarget‐directed ligands. This review lies in its holistic synthesis of EC's molecular underpinnings and therapeutic interventions, fused with an outlook on future directions including overcoming resistance mechanisms, biomarker discovery, and the potential of novel drug formulations.

## Graphical abstract

Potential strategies and potential targets for treating EC. Current the EC's&amp;#x000A0;research&amp;#x000A0;focuses on pivotal genes EGFR, HER2, TP53, CDKN2A, and APC, targeting key pathways in cell regulation and signaling. We highlight four novel therapeutic strategies: combined targeted therapy with chemotherapy, radiotherapy, immunotherapy, and multitarget approaches. Mechanistic insights into esophageal carcinogenesis underscore alterations in EGFR/ErbB, PI3K/AKT/mTOR, and Notch/Wnt/β‐catenin pathways, driving proliferative and survival signaling cascades.

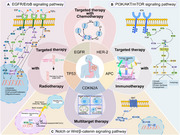

## 1 INTRODUCTION

Esophageal cancer (EC) ranks among the top eight most common cancers worldwide, particularly in China, where data from 2022 indicated 510,716 reported new cases and deaths.^1^ EC is primarily divided into esophageal squamous cell carcinoma (ESCC) and esophageal adenocarcinoma (EAC). ESCC is prevalent in East Africa and East Asian countries, typically occurring in the middle or upper part of the esophagus and sharing features with head and neck squamous cell carcinoma. EAC, more common in North America and Western Europe, usually arises in the lower esophagus with a risk factor of gastroesophageal reflux disease and shares a genomic profile with chromosomally unstable gastric adenocarcinoma.^2^ Clinically, according to the 2024 Chinese Society of Clinical Oncology (CSCO) guidelines, for patients with recurrent or metastatic ESCC, second line and above treatments are recommended with anlotinib, apatinib, and tertiary recommendation is carrelizumab + apatinib. For HER‐2 positive patients, trastuzumab is undoubtedly recommended. Despite advances in therapies targeting EGFR, VEGFR, HER‐2, and others, 5‐year overall survival (OS) rates for EC in China remain dishearteningly low at 15–25%.^3^ This grim outlook is primarily due to late diagnosis, rapid disease progression, and the complexity of its molecular pathogenesis.

Over recent decades, significant advances in cancer research have led to new therapies such as targeted therapy and immunotherapy, yet these advances have not substantially increased survival for many EC patients, partly due to intricate resistance mechanisms. In this review, we aim to dissect the current landscape of molecular targets in EC therapy, delving into the latest clinical trials, emerging treatment options, and promising research avenues. The focus is on exploring how novel therapeutic strategies and a multidisciplinary approach can potentially shift the treatment paradigm for EC.

The crux of this review pivots around the molecular pathogenesis of EC, emphasizing the pivotal role of key oncogenes, tumor suppressor genes, and epigenetic modifications. It underscores the urgent need for comprehensive molecular typing and the identification of effective biomarkers to refine patient selection for these therapies. By delving into the genetic and epigenetic mosaic that underlies EC, particularly focusing on key oncogenes and tumor suppressor genes. The review emphasizes the latest advancements in targeted drugs approved for clinical use, challenges in overcoming drug resistance, and the exciting potential of combination therapies that exploit the synergistic effects between different therapeutic modalities.

Moreover, this review elaborates on the revolutionary impact of immunotherapies, including the use of immune checkpoint inhibitors (ICIs) and novel drug formulations like antibody–drug conjugates (ADCs), offering new hope for EC management. It brings to light the intricacies of the tumor microenvironment (TME) and immune evasion mechanisms, presenting the base for a holistic approach that integrates targeted therapies, immunotherapies, and a profound comprehension of TME dynamics.

In conclusion, this review heralds a new era in the management of EC, suggesting that the fusion of molecular medicine, precision therapy, and immunotherapy could transform the clinical outcomes for patients with EC. By providing a comprehensive synthesis of current insights and future directions in EC research, it aims not only to advance the scientific discourse but also to ignite optimism for enhanced patient prognosis through innovation and precision medicine.

## 2 MOLECULAR PATHOGENESIS OF ESOPHAGEAL CARCINOMA

The understanding of molecular subtypes and driving factors of EC remains rudimentary, as technologies for identifying effective biomarkers or molecular typing are still underdeveloped. Therefore, traditional treatment modalities are still primarily used in clinical practice. Recent exploration of genetic and epigenetic profiles, including CCND1 and CDKN2A/B loci,^4^ is gradually deciphering EC's molecular pathogenesis. Exome sequencing reveals prevalent TP53 mutations in ESCC, alongside rare mutations in NOTCH1, NFE2L2, and CDKN2A^5^ suggesting transcriptome remodeling as a risk factor in ESCC development. Additionally, high‐throughput sequencing has linked the molecular classification of ESCC to the activation of specific cellular pathways, including those involved in the cell cycle and NRF2 signaling.^6^ Furthermore, the interaction between the ESCCAL‐1 gene and Galectin‐1z promotes cell cycle progression and EC cell proliferation. In the molecular pathogenesis of EAC, the transcription factor FOXM1 plays a crucial role in promoting tumor growth.^7^ FOXM1 facilitates tumor progression by inhibiting the infiltration of CD8⁺ T cells, a process mediated through the modulation of Th1 chemokine expression.^8^ This mechanism underscores the significant impact of cellular immunity on the dynamics of tumor development in EAC. Additionally, ERBB2 signal transduction serves as a critical upstream regulator of FOXM1, emphasizing its role in EAC pathogenesis.^9^

### 2.1 Genetic and epigenetic alterations

#### 2.1.1 Key oncogenes

Contemporary oncology research has identified the dysregulation of cell signaling pathways as a fundamental contributor to tumor development and progression. Significant attention has been paid to three crucial genes and proteins—EGFR, HER2, and TP53—and their substantial influence on cancer progression.

Epidermal growth factor receptor (EGFR) is a transmembrane protein composed of 1186 amino acids. Upon ligand binding, such as with epidermal growth factor (EGF) or transforming growth factor‐alpha (TGF‐α), EGFR undergoes dimerization.^10^ This dimerization activates multiple signaling pathways, including the PI3K–AKT–mTOR pathway involved in cell survival,^11^ the JAK/STAT pathway involved in immune regulation,^12^ and the MAPK/ERK pathway involved in cell proliferation.^13^ Excessive expression of EGFR enhances these pathways, boosting tumor cell proliferation and metastasis.^14^ Furthermore, EGFR also contributes to the TME in EC by increasing cellular reliance on the extracellular matrix (ECM), thereby promoting cancer cell growth and survival within the ECM.^15^ Concurrently, it contributes to increased PD‐L1 expression, suppressing immune system response against the tumor.^16^

Similarly, the HER2 gene, another critical oncogene, encodes a protein with tyrosine kinase activity.^17^ It is commonly highly expressed in EC, especially at the gastroesophageal junction (GEJ) cancer, ranging from 4.4 to 53.4%.^18^ HER2 activation triggers key signaling pathways, including PI3K/AKT/mTOR^19^ and RAS–RAF–MEK–ERK,^20^ which drive tumor cell proliferation, inhibit apoptosis, and influence tumor growth, survival, and metastasis.^21^, ^22^

The TP53 gene, one of the most critical tumor suppressor genes, produces the p53 protein that inhibits cell division or survival.^23^ TP53 mutations deactivate p53, leading to uncontrolled cell proliferation and invasion.^24^ Experimental studies have illustrated that TP53 mutations can accelerate tumorigenesis by interacting with the nuclease Mre11 and disrupting the Mre11–rad50–nbs1 complex, thereby inhibiting the activation of ataxia telangiectasia mutated.^25^ Moreover, some TP53 structural mutants, like R175H, have been linked explicitly to cancer development.^26^ Research on ESCC biopsy samples indicates that TP53 and NOTCH1 are the most frequently mutated genes,^27^ with TP53 mutations leading to increased ΔNP63 expression, thereby activating the EFNB1–EPHB4 influenced SRC/ERK/AKT signaling pathway. This activation is crucial in processes such as the epithelial–mesenchymal transition (EMT) and cell cycle progression, providing deep insights into ESCC pathogenesis.

#### 2.1.2 Tumor suppressor genes

The study of molecular pathology in oncology highlights the critical role of tumor suppressor genes in disease progression. Specifically, the CDKN2A and APC genes are central to cell cycle regulation and signaling pathways.

CDKN2A, located on chromosome 9, is part of the INK4 family and inhibits the cell cycle by regulating the pRb–E2F pathway, thereby blocking G1 to S phase progression and controlling cell proliferation.^28^ In EC, more than 60% of cases show a deletion of CDKN2A gene copies, which is a common genetic alteration observed in EC.^6^ Studies using EC organoids and single‐cell transcriptomics have shown that CDKN2A loss activates the CCL2–CCR2 axis, promoting tumor formation, immune evasion, and ESCC development.^27^

Another significant tumor suppressor gene, APC, is integral to the Wnt signaling pathway overseeing cell proliferation and differentiation. APC loss leads to abnormal cytoplasmic β‐catenin accumulation, which translocates to the nucleus and activates the Wnt signaling pathway, driving excessive cell proliferation and tumorigenesis.^29^, ^30^ APC mutations are relatively rare in ESCC, with a mutation rate of around 1.5%.^31^

#### 2.1.3 Epigenetic modifications

In cancer biology, epigenetic modifications, notably DNA methylation and histone modification, are pivotal in ESCC development. They regulate tumor initiation, progression, heterogeneity, drug resistance, and treatment response.

DNA methylation, either via the hypomethylation state or hypermethylation of specific CpG island promoters, plays a critical role in ESCC progression.^32^ It leads to either chromosomal instability or gene mutations and allelic losses, potentially inactivating tumor suppressor gene promoters.^33^ Contemporary studies are exploring DNA methylation's potential in subtyping and prognosticating EC, utilizing specific methylation sites^34^ to distinguish EC heterogeneity and as biomarkers for diagnosing EC state changes.^35^

Meanwhile, histone modification is also one of the important mechanisms regulating gene expression, involving processes like methylation, acetylation, phosphorylation, adenylation, ubiquitination, and ADP‐ribosylation. These modifications influence protein–DNA interactions, chromatin structure, and gene regulation.^36^, ^37^ Studies employing 4D proteomics have identified subtype‐specific phosphorylation and kinase–substrate network modifications in gastroesophageal junction adenocarcinoma, facilitating personalized treatments.^38^ Additionally, Kbu‐modified proteomics has uncovered how Kbu modifications enhance EC resistance and metastasis to therapies like 5‐fluorouracil, underscoring the complexity and importance of histone modifications in cancer research.^39^

### 2.2 Aberrant signaling pathways

#### 2.2.1 EGFR/ErbB signaling pathway

EGFR, a member of the ErbB family of receptor tyrosine kinases, activates key signaling pathways, including MAPK/ERK and PI3K/AKT, upon ligand binding and phosphorylation. These pathways are crucial for cell proliferation and survival. Notably, in ESCC, elevated EGFR and ERBB2 expression is significantly linked to transcription factors like KLF5, indicating high sensitivity to EGFs.^40^ In EAC, EGFR expression can reach up to 88%.^41^ EGFR activation also phosphorylates tyrosine residues that recruit adaptor proteins like GRB2 and Shc, triggering the PI3K pathway, often hyperactivated in EC.^42^ Additionally, EGFR activation initiates a kinase cascade (Raf, MEK, ERK), leading to ERK nuclear translocation and regulation of genes essential for cell proliferation and survival. Targeted therapies against the ErbB receptor, such as EGFR tyrosine kinase inhibitors (TKIs) and anti‐EGFR monoclonal antibodies, have delivered significant survival advantages in clinical applications. Nevertheless, acquired resistance, due to the activation of compensatory pathways and mutations emerging post first‐line treatment, has reduced tumor sensitivity to ErbB inhibitors, introducing a new challenge in targeted therapy.^43^

#### 2.2.2 PI3K/AKT/mTOR signaling pathway

The PI3K/AKT/mTOR pathway is activated when PI3K is stimulated by G protein‐coupled receptors. This interaction involves the recruitment of the p85 regulatory subunit to the plasma membrane, where it binds with the p110 catalytic subunit. This complex converts phosphatidylinositol 4,5‐bisphosphate to phosphatidylinositol 3,4,5‐trisphosphate, which then binds to the PH domain of Akt (protein kinase B), relocating Akt to the membrane and activating it. When AKT is activated, it can directly act on the Ser1448 site of mTOR, or act through the deactivation of tuberous sclerosis complex 2, thereby triggering its activation and the initiation of downstream pathways.^44^ This pathway not only promotes cell survival and proliferation, but also plays a role in promoting tumor formation, invasion, and metastasis by participating in angiogenesis.^45^ Unfortunately, the clinical utility of inhibitors targeting this pathway is currently limited due to significant treatment‐related toxicity. Future advancements in combination therapies or new drug development may enable the clinical application of inhibitors targeting this pathway.

#### 2.2.3 Notch or Wnt/β‐catenin signaling pathway

The Notch or Wnt/β‐catenin signaling pathway also plays important roles in the development of ESCC. The Notch signaling pathway has a key influence in regulating cell differentiation and proliferation,^46^ with approximately 38.3% of ESCC patients showing abnormalities in the Notch signaling pathway.^47^ This pathway involves the transport of the Notch receptor from the endoplasmic reticulum (ER) to the Golgi apparatus for proteolytic cleavage, followed by its movement to the cell surface where it interacts with cytoplasmic membrane ligands. This interaction leads to the release of the soluble Notch intracellular domain, which induces the formation of multiprotein–DNA complexes, thereby promoting the transcription of Notch target genes. Conversely, the Wnt/β‐catenin signaling pathway influences ESCC recurrence and progression by promoting EMT, angiogenesis, and metastasis. It regulates specific gene expressions, such as activating SAMD9^48^ and inhibiting PHGDH to control ESCC cell proliferation, cell cycle, and apoptosis, highlighting its therapeutic potential.^49^ Under normal physiological conditions, a degradation complex phosphorylates cytoplasmic β‐catenin, leading to its ubiquitination and proteasomal degradation. The binding of Wnt activates the FZD receptor, which recruits DVL to the plasma membrane. DVL's interaction with AXIN facilitates the receptor complex's recruitment and accumulation, resulting in cytoplasmic β‐catenin accumulation. β‐Catenin is then transported to the nucleus, where it binds with TCF/LEF and coactivators, leading to the activation of Wnt target genes. Mutations in tumor suppressor genes impair the destruction complex's activity, promoting tumor growth.^50^ Furthermore, research by Wei Wang and colleagues demonstrated that in ESCC, downregulation of the METTL3/YTHDF‐coupled epitope transcriptome via the APC gene negatively regulates the Wnt/β‐catenin pathway, thereby enhancing cell proliferation and tumor development.^29^

### 2.3 TME and immune evasion

#### 2.3.1 Role of cancer‐associated fibroblasts and immune cells

Cancer‐associated fibroblasts (CAFs) play a key role in the TME, secreting growth factors, cytokines, chemokines, extracellular vesicles (e.g., exosomes), and ECM components. These secretions categorize CAFs into tumor‐promoting and tumor‐suppressing types and facilitate the formation of restrictive barriers around lesion sites, which stiffen the ECM and obstruct cancer cell dissemination.^51^ The dynamically evolving TME, altered by ECM remodeling, creates resistance barriers that impede immune cell infiltration, thereby fostering a therapeutic resistance.^52^ Studies indicate that CAFs in ESCC significantly secrete exosomes that enhance cancer cell proliferation, invasion, and migration.^53^ Moreover, WNT2 expression in CAFs inversely correlates inversely with active CD8+ T‐cell presence, suggesting that WNT2‐secreting CAFs suppress antitumor T‐cell responses via the SOCS3/p‐JAK2/p‐STAT3 pathway.^54^ Additionally, the transition of mesenchymal stem cells to CAFs, often of epithelial origin, contributes to an immunosuppressive microenvironment by releasing factors like TGF‐β and matrix metalloproteinases (MMPs). This suppresses T‐cell function and supports cancer cell proliferation and therapy resistance.^55^, ^56^ CAFs also activate the PI3K/AKT pathway by binding to the integrin family, promoting cell proliferation, angiogenesis, and ECM reorganization. Secretion of MFGE8 and its interaction with integrins αVβ3/αVβ5 escalates tumor progression through further activation of the PI3K/AKT and ERK/AKT pathways.^57^ Conversely, inhibitory tumor‐type CAFs can negatively regulate tumor T cell responses by secreting immunosuppressive factors such as interleukin‐6 (IL‐6) and tumor necrosis factor‐alpha, or by promoting the recruitment of myeloid‐derived suppressor cells. These actions can effectively exert negative regulation against the tumor T cell response,^54^ leading to immune evasion within the TME. This immunosuppressive state allows tumors to evade host immune surveillance, thereby promoting tumor growth and metastasis. Additionally, CAFs modulate B‐cell activation and antibody production by altering cytokine levels, which impacts the humoral immune responses.^56^ They also encourage M2 phenotype polarization in macrophages, enhancing tumorigenic conditions.^58^ During cancer treatment, CAFs may release protective factors that reduce the efficacy of chemotherapy (CT), immunotherapy, and targeted therapy. Notably, CAFs induce PD‐L1 expression in ESCC cells, highlighting a potential immunotherapy target.^59^ Single‐cell RNA sequencing has shown that AXNA1's interaction with FPR2 on fibroblasts inhibits phosphorylation pathways, keeping fibroblasts quiescent and preventing their transformation into tumor‐promoting myofibroblasts.^60^, ^61^ This underscores the significance of the ANXA1/FPR2 signaling pathway as a therapeutic target in ESCC.

#### 2.3.2 Angiogenesis and hypoxia

Vascular endothelial growth factor (VEGF) and its receptors, VEGFR‐1 and VEGFR‐2, are principal regulators of angiogenesis, facilitating cell proliferation, migration, and survival.^62^ In solid tumors such as EC, hypoxic conditions stabilize hypoxia‐inducible factor‐1α (HIF‐1α), enhancing VEGF gene transcription and expression on tumor cells.^63^ Additionally, hypoxia accelerates the progression of ESCC and potentially promotes metastasis through activation of the Wnt/β‐catenin pathway^64^ via TCF4/TCF7L2 or by influencing the SP1 gene.^65^ Under these conditions, the expression of TMTC3 is markedly increased on the surfaces of ESCC cells. TMTC3 interacts with the Bateman domain of IMPDH2 to generate GTP, boosting the activity of the Rho GTPase/STAT3 pathway and further regulating VEGFA expression.^66^ Studies also suggest that hypoxic environments enhance tumor angiogenesis by overexpressing eIF5A2, which activates the HIF‐1α‐mediated signaling pathway,^67^ thus promoting tumor growth and dispersal. Moreover, exosomes containing Circ‐ZNF609 significantly contribute to hypoxia‐driven ESCC metastasis.^68^

#### 2.3.3 Immune checkpoints and immune evasion mechanisms

In the immune system, immune checkpoints are important molecules that regulate immune responses and maintain immune tolerance. In EC, the most prevalent checkpoint is the PD‐1/PD‐L1 axis, where the interaction of PD‐1 on T cells and PD‐L1 on tumor cells results in T cell dysfunction and apoptosis, enabling tumor cells to evade immune surveillance.^69^ Clinically, the application of PD‐1/PD‐L1 ICIs such as Nivolumab and Pembrolizumab has significantly prolonged the OS of patients with EC, greatly changing the first‐line treatment options for patients.^70^ In addition, CTLA‐4 is another significant immune checkpoint; it binds to B7 molecules, competitively inhibiting the early activation of T cells.^71^ In EC patients, increased CTLA‐4 expression is closely associated with tumor immune escape. Studies have shown that targeted therapy of CTLA‐4 could significantly improve the survival rate of patients with EC.^72^ Recent research efforts have focused on immune checkpoints such as TIM‐3, LAG‐3, and T cell immunoreceptor with Ig and ITIM domains (TIGIT) to unveil their roles in EC through various mechanisms. The elevated expression of these checkpoints in EC correlates with poor prognoses. By analyzing mRNA expressions in 51 ESCC tissues, Mahmoudian et al.^73^ uncovered a strong correlation among EMT, the TME, and the expression of multiple immune checkpoints and EMT‐related genes in ESCC. Therefore, the expression levels of immune checkpoints can also serve as biomarkers for predicting the prognosis of patients with EC.

Of course, tumor cells can also achieve immune escape through various other mechanisms, including the upregulation of immune checkpoint expression, secretion of immune‐suppressive factors, and alteration of the TME. EC cells can promote immune escape by overexpressing inhibitory molecules (e.g., PD‐L1/2, VISTA)^74^, ^75^ or inciting immune cells to express inhibitory proteins (such as PD‐1, CTLA‐4,^76^ T‐cell immunoglobulin and mucin‐domain containing‐3 (TIM‐3),^77^ and lymphocyte‐activation gene 3 (LAG‐3),^78^ which trigger inhibitory signaling in effector T cells. Additionally, tumor cells can secrete various immunosuppressive factors, like TGF‐β and IL‐10, reducing T and natural killer (NK) cell activity and fostering an immunosuppressive TME.^79^ Concurrently, EC cells can modify the TME to favor tumor growth. For instance, recruiting regulatory T cells (Tregs) heightens immunosuppressive effects while diminishing effector T cell numbers and function.^80^ In ESCC, CAFs expressing fibroblast activation protein secrete IL‐6 and CCL2, negatively regulating antitumor mechanisms and inducing M2 macrophage‐like polarization to promote an immunosuppressive TME.^81^ High CCL2 expression on ESCC cells induces monocytes and tumor‐associated macrophages, advancing immune evasion.^82^ Wu et al.^83^ highlighted that elevated MAGE‐C3 expression in ESCC facilitates EMT and immune evasion, enhancing metastasis, and suggesting MAGE‐C3 as a prognostic marker and therapeutic target. Further genomic sequencing, particularly in ESCC patient samples, revealed the pivotal role of Hippo/YAP signaling pathway overactivation in ESCC carcinogenesis and progression.^84^ YAP is shown to regulate CD24 expression by binding to TEAD and activating the CD24 promoter, aiding tumor cell evasion from macrophage clearance.^85^

### 2.4 Mechanisms of invasion and metastasis

#### 2.4.1 Epithelial–mesenchymal transition

EMT and cellular proliferation are key in epithelial cancer progression. In ESCC, insights from single‐cell transcriptomics have identified a notable pattern; patients whose ESCC is categorized by an EMT gene set with pronounced EMT features tend to have less favorable prognoses. These EMT traits primarily arise early in the tumor's development, prior to lymph node metastasis, accelerating ESCC progression.^86^ Subsequent investigations utilizing RNA sequencing of 225 ESCC tissue samples elucidated the mechanism of PURα in EMT induction through its interaction with the Snail2 promoter region.^87^ This discovery offers a novel understanding of EMT's influence on ESCC advancement. In addressing the EMT pathway, various investigations have aimed to identify new intervention strategies. Notably, some studies focus on suppressing key EMT transcription factors by modulating Rab11–FIP1^88^ or manipulating TWIST1 expression to increase E‐cadherin and vimentin levels, thus influencing the EMT process.^89^, ^90^ These findings highlight EMT's crucial role in ESCC initiation and progression and suggest promising therapeutic approaches for controlling ESCC via EMT regulation.

#### 2.4.2 Matrix metalloproteinases

MMPs, particularly MMP2 and MMP9, are crucial zinc‐dependent endopeptidases that facilitate cancer cell migration.^91^ Studies have shown that a monoclonal antibody targeting PTK7 can diminish cancer cell invasion by decreasing MMP9 secretion.^92^ Additionally, protein‐protein interaction experiments have demonstrated that the LCN2/LOXL2/MMP9 trimeric complex enhances ESCC cell migration and invasion, accelerating tumor growth and malignant progression in vivo.^93^ MMP3 is also a key regulatory enzyme involved in the invasion and migration processes of ESCC.^94^

Further research has identified MMP13 as an enzyme capable of degrading the ECM, increasing the invasiveness of ESCC through interactions with CD44 and TWIST1, and it is recognized as a significant regulatory factor in the EMT process of ESCC. MMP13 functions as both a diagnostic marker and a potential therapeutic target.^95^ In EAC, significant expression of APE1 (apurinic/apyrimidinic endonuclease 1) has been shown to regulate MMP‐14, activating MMP‐2 and leading to redox‐dependent ECM degradation, thus affecting the invasive capacity of cancer cells.^96^

## 3 MOLECULAR TARGETS AND THERAPEUTIC STRATEGIES

### 3.1 Targeted therapy

Targeted therapy is a precision medicine strategy based on specific molecular mechanisms, playing an increasingly important role in the treatment of EC. Enhanced understanding of EC's molecular landscape has identified several key targets, including EGFR, HER2, and VEGF, which are closely linked to the disease progression and patient prognosis. Targeted therapies inhibiting these pathways can effectively slow EC progression and improve outcomes, particularly in local control rates, progression‐free survival (PFS), and OS. However, the application of targeted drugs in EC also faces challenges, and the management of efficacy and side effects is one of the urgent issues to be addressed (Tables 1 and 2).

**TABLE 1:** A summary of clinical studies on targeted therapy for esophageal cancer.

	Trial	Phase	Number	Stage	Type	Line	*N*	Intervention	Result
Anti‐EGFR	/	II	NCT05221658	Local advance or advanced	EC	First line vs. non‐first line	49	HLX07 + Serplulimab + CT vs. HLX07	ORR 55.2 vs. 23.1% DCR 72.4 vs. 38.5% PR 55.2 vs. 23.1% SD 17.2 vs. 15.4% PD 13.8 vs.46.2% PFS NE vs. 1.5 months TRAEs 96.7 vs. 68.4%
	/	II	/	Advanced	ESCC	Second line	41	Camrelizumab + Nimotuzumab	ORR 36% mOS 12.62 months mPFS 9.17 months DCR 81% TRAEs 60%
	POWER^97^	III	NCT01627379	Advanced	G/GEJC	First line	142	Panitumumab + CT vs. CT	mOS 10.2 vs. 9.4 months ORR 37.0%
Anti‐VEGFR	/^98^	Ib	NCT03671265	Local advanced	ESCC	First line	20	Camrelizumab + CRT	OS 8.2–28.5 months PFS 4.0–28.5 months 12‐month OS 85% 24‐month OS 69.6% 12‐month PFS 80.0% 24‐month PFS 65.0%
	/	II	NCT03917966	Local advanced	ESCC	Neoadjuvant	24	Camrelizumab + Apatinib	R0 100% ORR 50% pCR 10.5% MPR 42.1% Down‐staging 68.4%
	/	II	/	Local advanced	ESCC	Neoadjuvant	41	Apatinib + CT	1‐year OS 95% 2‐year OS 95% 1‐year DFS 85% 2‐year DFS 82% pCR 23.6% MPR 39.5%
	/^99^	II	NCT04345783	Advanced	G/GEJC	First line	24	Camrelizumab + Apatinib + CT	ORR 29.2% mPFS 6.5 months 3–4 TRAEs 25.0%
	CAP 02^100^	II	NCT03736863	Advanced	EC	Second line or above	33	Camrelizumab + Apatinib	mOS 15.8 months PR 41.18% SD 52.94% ORR 41% TRAEs 67%
	ALTER‐E006^101^	Retrospective	ChiCTR2300070777	Advanced	ESCC	Second line or above	96	Anlotinib + ICIs	ORR 21.9% DCR 67.7% mOS 10.97 months mPFS 6.31 months TRAEs 69.8% ≥3 TEAEs 3.1%
	ALTER‐E003	II	NCT05038813	Advanced	ESCC	First line	46	Anlotinib + TQB2450	ORR 69.6% DCR 91.3% CR 2.2% PR 58.7% SD 30.4% mPFS 15.44 months
	TQB2450‐II‐13	II	NCT05013697	Local advanced or advanced	ESCC	First line	50	Anlotinib + Bemosubezumab + CT	ORR 82.2% DCR 100% 3–4 TRAEs 6%
	/	II	NCT04471480	Advanced	EC	First line	69	Anlotinib + ICIs + CT vs. ICIs + CT vs. CT	mPFS 13.4 vs. 7.2 vs. 4.8 months ORR 90 vs. 43.3 vs. 23.3%
	/^102^	II	NCT02649361	Advanced	ESCC	Second line or above	165	Anlotinib vs. placebo	mPFS 3.02 vs. 1.41 months
	/	Ib/II	NCT05024812	Advanced	E/GEJ	First line	17	Triplimab + Fruquintinib + CT	PR 56.25% SD 43.75% ORR 56.3% DCR 100% mPFS 9.3 months
	EDGE‐Gastric	II	NCT05329766	Advanced	E/GEJ	First line	41	Domvanalimab + CT	ORR 59% PFS 77.%
	KEYVIBE‐005^103^	II	NCT05007106	Advanced	EC	First line	40	Vibostolimab + Pembrolizumab	ORR 53% DCR 85% mDOR 13.9 months mPFS 10.4 months mOS 18.0 months TRAE 95% ≥3 TEAEs 73%
	SKYSCRAPER‐08	III	NCT04540211	Advanced	ESCC	First line	464	Tiragolumab + Atezolizumab + CT vs. placebo + CT	CR 11.5 vs. 3.2% PR 48.2 vs. 42.3% mDoR 7.1 vs. 4.3 months mOS 15.7 vs. 11.1 months mPFS 6.2 vs. 5.4 months TRAEs 98.2%
Anti‐Claudin18.2	/	I	NCT04495296	Advanced	G/GEJ	First line	12	TST001 + CT	PR 33.3% SD 25.0% PD 8.3%
	/	II	NCT03925974	Advanced	G/GEJ	Second line or above	45	TST001 (HER2 high vs. HER2 low)	ORR 56 vs. 14% DoR 9.7 vs. 6.2 months mPFS 8.3 vs. 1.4 months mOS 16.3 vs. 9.6 months
	/^104^	I	NCT03528629	Advanced	G/GEJ	Second line or above	18	Zolbetuximab (600 mg/m^2^ vs. 1000 mg/m^2^Q3W)	SD 64.7% PD 35.3% DCR 35.3% mOS 4.4 vs. 6.4 months mPFS 2.6 vs. 1.7 months
	MONO^105^	IIa	NCT01197885	Advanced	G/GEJ	Second line or above	54	Zolbetuximab	ORR 9% SD 14% PR 9.3% TRAEs 81.5%
	SPOTLIGHT^106^	III	NCT03504397	Local advanced or advanced	G/GEJ	First line	556	Zolbetuximab + CT vs. placebo + CT	mPFS 10.61 vs. 8.67 months ≥3 TRAEs 87% vs. 78%
	GLOW^107^	III	NCT03653507	Local advanced or advanced	G/GEJ	First line	507	Zolbetuximab + CT vs. placebo + CT	mPFS 8.21 vs. 6.80 months mOS 14.39 vs. 12.16 months ≥3 TRAEs 72.8% vs. 69.9%
Anti‐FGFR2b	FIGHT^71^	II	NCT03694522	Advanced	G/GEJ	First line	155	Bemarituzumab + CT vs. placebo + CT	mPFS 9.5 vs. 7.4 months mOS 19.2 vs. 13.5 months
Anti‐PI3K	EPOC1303^108^	II	UMIN 000011217	Advanced	ESCC	Second line or above	42	BKM120	SD 47.62% cPR 4.76% mPFS 2.3 months mOS 9.0 months DCR 51.2%
Anti‐ MMP‐9	/^109^	Ib	NCT02862535	Advanced	G/GEJC	First line	16	Andecaliximab + CT	ORR 73% mPFS 11.9 months
Anti‐ B7‐H6/CD3 T cell adapter	BEAR^110^	II	NCT04839471	Advanced	ESCC	Second line or above	19	BI‐75409 + Afatinib	PR 36.84% SD 36.84% ORR 36.8% mPFS 6.8 months
CAR‐T	CT041‐ST‐01^111^	Ib/II	NCT04581473	Advanced	G/GEJ	Second line or above	192	CT041	PR 57.1% SD 14.3% PFS 5.6 months OS 10.8 months
BsAb: PD‐1, CTLA‐4	COMPASSION‐04^112^	Ib/II	CTR20182027	Advanced	G/GEJC	First line	98	Cadonilimab + CT	mDOR 13.73 months mPFS 8.18 months mOS 17.48 months ORR 52.1% CR 4.3% PR 47.9%
	AK104‐IIT‐014	II	NCT05522894	Advanced	ESCC	First line	22	Cadonilimab + CT	PR 59.1% SD 9.1% ORR 86.7% DCR 100%
	/	Ib	/	Advanced	ESCC	First line	25	KN046 + CRT	ORR 41.7% mPFS 7.8 months mOS 15.9 months
BsAb: PD‐L1, TGF‐β	/^113^	I	NCT02699515	NE	ESCC	Second line or above	30	Bintrafusp alfa	TRAEs 63.3% ORR 20.0% mOS 11.9 months
MTDL: AXL, VEGFR2, FLT3	/	Ib/II	NCT05260385	Advanced	ESCC	Second line or above	133	KC1036	mPFS 4.2 months ORR 29.2% DCR 83.3%
/	TQB2450‐II‐13	II	/	Advanced	ESCC	First line	214	ICIs + CT vs. ICIs + anti‐vascular vs. ICIs + CT + anti‐vascular	mOS 9.1 vs. 10.0 vs. 11.5 months mPFS 4.3 vs. 4.3 vs. 5.3 months

*Data sources*: Clinical registration website (www.clinicaltrials.gov) (www.chictr.org.cn), ASCO meeting (www.asco.org), ESMO meeting (www.esmo.org).

Abbreviations: *N*, the number of participants; ESCC, esophageal squamous cell carcinoma; EAC, esophageal adenocarcinoma; EC, esophageal cancer; CT, chemotherapy; CR, complete response; CRT, chemoradiotherapy; DCR, disease control rate; ICI, immune checkpoint inhibitors; GEJ, gastroesophageal junction; mOS, median overall survival; mPFS, median progression‐free survival; ORR, objective response rate; pCR, pathological complete response; PR, partial response; R0, complete surgical resection; SD, stable disease; TRAEs, treatment‐related adverse events; MPR, major pathological response.

**TABLE 2:** A summary of ongoing clinical studies on targeted therapies for esophageal cancer.

	Trial	Phase	Number	Stage	Type	Line	*N*	Intervention	Primary endpoint	Main result
Anti‐EGFR	/	I	NCT06048913	Local advanced	EC	First line	45	Nimotuzumab + CRT	OS	Enrolling by invitation
	ESCC‐ALTRK	II	NCT05818982	Advanced	ESCC	Second line or above	72	Afininib vs. CT	PFS	Recruiting
	/	II	NCT01034189	Local advanced	ESCC	Adjuvant	62	Cetuximab + CRT	Clinical response rate	Unknown status
	/	II	NCT06374888	Advanced	G/GEJC	First line	28	Neratinib + CT	ORR	Not yet recruiting
Anti‐VEGFR	/	II	NCT05866510	Advanced	EC	Second line or above	47	Anlotinib + Utidelone	ORR	Recruiting
	/	II/III	NCT03285906	Advanced	G/GEJC	Second line	30	Apatinib	PFS	Unknown status
	/	II	NCT03030937	Advanced	G/GEJC	Second line	74	Apatinib + CT vs. CT	PFS, AEs	Unknown status
	LEAP‐014^114^	III	NCT04949256	Advanced	ESCC	First line	862	Lenvatinib + Pembrolizumab + CT	DLT, AEs, OS	Recruiting
Anti‐TIGIT	Tigit 203	II	NCT04732494	Advanced	ESCC	Second line	280	BGB‐A1217 + Tislelizumab vs. placebo + Tislelizumab	ORR, OS	Recruiting
Anti‐HDAC	/	II	ChiCTR2400085302	Local advanced	ESCC	Neoadjuvant	78	Chidamide + Sintilimab	pCR	Recruiting
Anti‐FGFR2B	FORTITUDE‐101	III	NCT05052801	Advanced	G/GEJC	First line	516	Bemarituzumab + CT vs. placebo + CT	OS	Active, not recruiting
	FORTITUDE‐102	III	NCT05111626	Advanced	G/GEJC	First line	528	Bemarituzumab + Nivolumab + CT vs. placebo + Nivolumab + CT	TRAEs	Recruiting
Anti‐TIM‐3	TQB2618‐AK105‐IB‐03	I/II	NCT05834543	Advanced	ESCC	First line	75	TQB2618 + Penpulimab + CT vs. Penpulimab + CT vs. TQB2618 + Penpulimab + TQB3617	PFS, ORR	Not yet recruiting
Anti‐CDK4/6	SCOG007	I	NCT05927844	Advanced	ESCC	Second line or above	20	XH‐30002 + Afatiinb	SAEs, severity of TEAE and SAE	Not yet recruiting
BsAb: PD‐1, LAG‐3	MAHOGANY^115^	II/III	NCT04082364	Advanced	G/GEJC	First line	82	Margetuximab + Retifanlimab + CT vs. Margetuximab + Tebotelimab + CT	ORR, OS, safety	Active, not recruiting
BsAb: PD‐1, CTLA‐4	/	II	NCT05990231	Advanced	ESCC	Second line	35	Cadonilimab + Anlotinib	ORR	Recruiting
Anti‐Claudin18.2	/	I	NCT06353152	Advanced	G/GEJC	First line	12	Claudin18.2‐targeted chimeric antigen receptor T cell injection	DLT, AEs	Recruiting
ADC: Nectin‐4	/	I/II	NCT06474468	Advanced	EC	Second line	148	SHR‐A2102 + Adebrelimab	DLT, ORR	Recruiting
ADC: Trop‐2	/	II	NCT06329869	Advanced	ESCC	Second line	35	Sacituzumab govitecan	ORR	Not yet recruiting
ADC: EGFR, HER2	/	III	NCT06304974	Advanced	ESCC	Second line or above	488	BL‐B01D1 vs. CT	PFS, OS	Recruiting
MTDL: FGFR, CSF1R, KDR	SYSA1501‐010	I/II	NCT06577376	Advanced	G/GEJC	Second line	252	DP303c + Simmitinib vs. DP303c + CT vs. CT	DLT, AEs, ORR, SAEs	Not yet recruiting
MTDL: AXL, VEGFR2, FLT3	KC1036‐III‐01	III	NCT06194734	Advanced	ESCC	Third line	490	KC1036 vs. CT	OS	Recruiting

*Data sources*: Clinical registration website (www.clinicaltrials.gov) (www.chictr.org.cn).

Abbreviations: *N*, the number of participants; ADC, antibody–drug conjugate; AXL, anexelekto; BsAb, bispecific antibodies; CRT, chemoradiotherapy; CSF1R, colony stimulating factor 1 receptor; CT, chemotherapy; CTLA‐4, cytotoxic T‐lymphocyte‐associated protein 4; DCR, disease control rate; DLT, dose limiting toxicity; EC, esophageal cancer; ESCC, esophageal squamous cell carcinoma; EGFR, epidermal growth factor receptor; FGFR, fibroblast growth factor receptor; FLT3, FMS‐like tyrosine kinase 3; GEJ, gastroesophageal junction; HER2, human epidermal growth factor receptor 2; KDR, kinase domain‐containing receptor; LAG‐3, lymphocyte activation gene‐3; MTDL, multitarget‐directed ligands; ORR, objective response rate; OS, median overall survival; pCR, pathological complete response; PFS, median progression‐free survival; TRAEs, treatment‐related adverse events; VEGFR, vascular endothelial growth factor receptor.

EGFR amplification and overexpression are commonly observed in EC, with mutations in this receptor significantly contributing to tumor progression and invasion. Gefitinib, an EGFR inhibitor, has been shown to induce dose‐dependent growth arrest in cancer cells.^116^ A Phase II study indicated that Gefitinib showed limited activity in the second‐line treatment of advanced EC patients, with 2.8% reaching PD, 27.8% achieving SD, and 47.2% showing PR. In subgroup analysis, Gefitinib also demonstrated a prognostic improvement advantage for patients with high EGFR expression.^117^ This finding was similarly observed in another Phase III study that did not selectively enroll patients. However, this result may not be applicable to our Asian population due to ethnic and regional differences.^118^ Clinical studies have demonstrated that Cetuximab, when combined with FOLFOX CT and radiotherapy, was both effective and well‐tolerated in treating locally advanced EC, achieving median OS (mOS) of 21.6 months and median PFS (mPFS) of 11.3 months.^119^ The Phase II LEOPARD‐2 trial showed that combining Cetuximab with CCRT for unresectable EC trends toward improved PFS and metastasis‐free survival (MFS).^120^ But, since the sample sizes were all below 100 and the Phase III trials have not been confirmed, the reliability remains questionable. Additionally, a Phase IB/II study found that adding Cetuximab increased the R0 resection rate, enhanced pathologic complete response (pCR) rates (55 vs. 20%), and improved local control rates (96 vs. 74%). In studies on neoadjuvant therapy for resectable EC patients, compared with the CCRT group, the Cetuximab + CCRT group significantly improved local control rate (*p* = 0.017), although there was no significant difference in PFS and OS.^121^ Similarly, in the early RTOG 0436 Phase III clinical trial, its combination with chemoradiation did not show benefits to OS.^122^ The E2205 trial was also prematurely terminated due to the toxicity side effects of Cetuximab.^123^ Recent clinical datas suggest that while Cetuximab may effectively improve local control rates, its impact on OS and PFS is limited, and its adverse effects necessitate careful management. Besides, from both the perspectives of sample size and the participant population of Asian descent, these results hold significant value for us. Nimotuzumab for locally advanced patients, in combination with chemoradiotherapy, has been shown to increase the pCR (62.3 vs. 37.0%, *p* = 0.02) and is beneficial for improving patients' quality of life.^124^ In cases of unresectable ESCC, the addition of Nimotuzumab to concurrent chemoradiotherapy resulted in improved objective response rates (ORR), as well as extended PFS and OS, as evidenced by the Phase III NXCEL1311 trial.^125^ However, some studies have indicated that while the Nimotuzumab plus CCRT group did not show significant differences in OS and PFS compared with the CCRT group, there was a notable reduction in the risk of brain metastases among patients. Overall, the benefits of Nimotuzumab, whether in terms of PFS, OS, or safety, are surprisingly positive. Additionally, Icotinib, a highly selective EGFR inhibitor, has demonstrated substantial antitumor activity in advanced ESCC patients with EGFR overexpression or amplification, achieving median PFS and OS of 52 and 153 days, respectively.^126^ A Phase II randomized clinical trial further revealed that combining Icotinib with radiotherapy provided survival benefits and maintained manageable safety in elderly patients with unresectable ESCC.^127^

HER2 is an important molecular target for EC treatment. For patients with HER2‐positive advanced E/GEJ, anti‐HER2 therapies have been established as frontline treatment options. Additionally, neoadjuvant therapies that incorporate anti‐HER2 agents have emerged as effective strategies for operable HER2‐positiveE/GEJ adenocarcinomas. A Phase II clinical trial assessed the ADC drug DS‐8201, containing Trastuzumab, for the second‐line treatment efficacy in patients with HER2‐positive esophagus or gastric junction adenocarcinoma, showing significant improvement in OS (12.5 m vs. 8.4 m, *p* = 0.01).^128^ Moreover, ADC drugs related to Trastuzumab, such as ARX788, have also come into our view and shown promising prospects.^129^ Perioperative use of trastuzumab in patients with resectable HER2‐positive gastric or GEJ cancer, particularly those with extensive lymph node metastasis, has shown both feasibility and effectiveness.^130^, ^131^ However, studies have indicated that Trastuzumab did not improve PFS (3.2 m vs. 3.7 m) for HER2‐positive advanced gastric and GEJ cancer patients, and this study was primarily conducted in Japan, with the number of subjects far exceeding those of previous studies.^132^ Lapatinib, a dual inhibitor of EGFR and HER2, in the TRIO‐013/LOGiC Phase III clinical trial, found that the addition of Lapatinib to CapeOx did not increase OS for HER2‐amplified gastroesophageal adenocarcinoma (GEA) patients (12.2 m vs. 10.5 m, *p* = 0.91).^133^ Additionally, in a Phase II trial, KN026, an anti‐HER2 agent, exhibited a favorable safety profile in patients with both high and low levels of HER expression, achieving ORR of 56 and 14%, respectively.^134^

VEGF expression is closely related to the progression and prognosis of EC, and the VEGF/VEGFR signaling pathway has become one of the effective targets for EC. Trials have shown that adding Bevacizumab to capecitabine + cisplatin for advanced gastric cancer patients did not improve the AVATAR trial's outcome, nor did it bring a significant improvement in OS.^135^ In contrast, after CRT in locally advanced non‐small cell lung cancer (LA‐NSCLC) showed significant improvements in PFS and OS, underscoring the potential benefits of combining antiangiogenic agents with immunotherapy in LA‐NSCLC.^136^ Yet, studies have indicated that Bevacizumab might be related to impaired wound healing in patients, although the enrollment did not surpass 100 individuals.^137^ Since Ramucirumab combined with paclitaxel has shown significant benefits for OS,^138^ this regimen has become one of the standard second‐line treatment options for advanced gastric or GEJ adenocarcinoma. The feasibility of the nab‐PTX plus Ramucirumab regimen was also proposed in the B‐RAX trial.^139^ Similarly, in trials comparing the FOLFIRI‐Ram regimen with the paclitaxel and Ramucirumab regimen, the RAMIRIS trial demonstrated the feasibility and effectiveness of the FOLFIRI‐Ram, proving its good PFS and safety for advanced GEA,^140^ though there were dissenting views as well.^141^ The RAINFALL trial indicated that the addition of Ramucirumab to platinum‐based CT with fluoropyrimidine as a first‐line treatment for patients with metastatic gastric or GEA is not recommended.^142^ Additionally, Ramucirumab monotherapy also demonstrated certain clinical activity and controllable safety in patients whose disease progressed after first‐line CT.^143^ Overall, Ramucirumab has demonstrated encouraging results across various large‐scale studies. In the realm of neoadjuvant therapy for ESCC, the combination of Anlotinib with Sintilimab and CT has shown improvements in pCR. However, this regimen did not significantly outperform immunotherapy combined with CT alone, indicating that the role of antiangiogenic agents in neoadjuvant settings for ESCC remains unclear.^49^ Meanwhile, the combination of Anlotinib with TP in the first‐line treatment of advanced ESCC showed enduring clinical benefits and controllable safety (mPFS 8.38 m, mOS 18.53 m).^144^

### 3.2 Immunotherapy

In recent years, ICIs have demonstrated promising clinical efficacy in patients with advanced EC. Clinical studies have shown that ICIs, as a part of second‐line treatment options, significantly prolong the OS and PFS of patients and improve the clinical response rate, especially when used in combination with CT.^145^ Regarding safety, the incidence of immune‐related adverse events (irAEs) has undeniably increased to some extent, but it remains relatively low, maintaining an optimistic tolerance profile. Immunotherapy's role in EC has expanded from advanced stages to include locally advanced and early‐stage disease, transforming the therapeutic landscape (Tables 3, 4, 5, 6, 7). For first‐line treatment of unresectable EC, recent findings from the RATIONALE‐306 study, presented by the CSCO in 2024, confirmed the survival benefits of Tislelizumab in a cohort of 649 patients with advanced EC. The study reported a median PFS of 7.3 months compared with 5.6 months, and a median OS of 17.2 months versus 10.6 months for the control group.^146^ Similarly, the Phase III GEMSTONE‐304 study on Sugemalimab with 540 ESCC patients also achieved both PFS and OS endpoints. Compared with CT alone, the combination of Carrelizumab with CT also showed an advantage in pCR rates (15.4 vs. 4.7%), indicating significant breakthroughs in immunotherapy for EC. Furthermore, to address the challenge of immune resistance, numerous studies are exploring the efficacy of combining immunotherapy with other treatments. For instance, the SKYSCRAPER‐08 study outcomes with 461 ESCC patients indicated that the Tiragolumab + Atezolizumab + CT group significantly improved PFS (6.2 m vs. 5.4 m) and OS (15.7 m vs. 11.1 m) compared with the placebo + CT group. The PALACE‐1 study, leveraging the synergistic effect between immunotherapy and radiotherapy, applied Pembrolizumab in combination with chemoradiotherapy as a neoadjuvant treatment for ESCC, achieving significant advantages in pCR (55.6%).^147^

**TABLE 3:** A summary of clinical studies on neoadjuvant immunotherapies for esophageal cancer.

Trial	Phase	Number	Stage	Type	Line	*N*	Intervention	Result
/	Foresight	ChiCTR2000040330	Local advanced	ESCC	Neoadjuvant	250	Camrelizumab + NCT vs. NCT	R0 100% PCR 27.8 vs. 10.0% MPR 43.3 vs. 21.7%
/	II	NCT02962063	Local advanced	EAC	Neoadjuvant	36	Durvalumab + NCRT + surgery	pCR 24.0% MPR 67.0%% 12‐month OS 92% 24‐month OS 85% 12‐month PFS 81% 24‐month PFS 71% 12‐month DFS 82% 24‐month DFS 78%
/	II	NCT05323890	Local advanced	ESCC	Neoadjuvant	19	Tislelizumab + NCRT	R0 100% PCR 50.0% MPR 72.2%
SCALE‐1^148^	Ib	ChiCTR2100045104	Resectable	ESCC	Neoadjuvant	23	Toripalimab + NCRT	pCR 55% MPR 80%
PALACE‐1^147^	Ib	NCT03792347	Resectable	ESCC	Neoadjuvant	20	Pembrolizumab + NCRT	pCR 55.6% ≥3 TRAEs 65.0%
TRAP^149^	I/II	NCT02120911	Resectable	E/GEJ	Neoadjuvant	40	Pertuzumab + Trastuzumab + NCRT	R0 100.0% pCR 34% 3y PFS 57% OS 71%
PANDA^150^	II	NCT03448835	Resectable	E/GEJ	Neoadjuvant	20	Atezolizumab + CT	MPR 70% pCR 45% R0 100%
PERFECT^151^	II	NCT03087864	Resectable	EAC	Neoadjuvant	40	Atezolizumab + NCRT	R0 83% pCR 25%
/^152^	II	ChiCTR2000029807	Resectable	ESCC	Neoadjuvant	47	Camrelizumab + NCT	R0 100% pCR 64.3% MPR 33.3% ORR 80.0% 1‐year DFS 97.6% 1‐year OS 97.6% 2‐year DFS 92.3% 2‐year OS 97.6%
GASTO1056^153^	II	ChiCTR2000028900	Resectable	ESCC	Neoadjuvant	23	Camrelizumab + NCT	pCR 25.0% MPR 50.0% R0 100.0% ORR 90.5% DCR 100%
SIN‐ICE^154^	II	NA	Resectable	ESCC	Neoadjuvant	23	Sintilimab + NCT	R0 94.1% pCR 35.3% MPR 52.9% ≥3 AE 30.4%
KEEP‐G 03^155^	II	ChiCTR1900026593	Resectable	ESCC	Neoadjuvant	30	Sintilimab + NCT	3‐4 TRAEs 36.7% pCR 20.0% MPR 50.0%
ESONICT‐1^156^	II	ChiCTR2100045659	Resectable	ESCC	Neoadjuvant	30	Sintilimab + NCT	ORR 67.0% pCR 21.7% MPR 52.2% R0 100.0%
SCCH‐TS2107^157^	II	NCT05189730	Resectable	ESCC	Neoadjuvant	20	Tislelizumab + NCRT	R0 100.0% pCR 50.0% MPR 72.2%
TD‐NICE^158^	II	NA	Resectable	ESCC	Neoadjuvant	45	Tislelizumab + NCT	R0 80.5% pCR 50.0% MPR 72.0% 3–4 TRAEs 42.2%
CRISEC^159^	II	NCT04776590	Resectable	ESCC	Neoadjuvant	26	Tislelizumab + NCRT	R0 100% pCR 46.7% MPR 86.7% ORR 60% DCR 100% 1–2 TRAEs 88.9%
/	II	ChiCTR2100051599	Resectable	ESCC	Neoadjuvant	26	Tislelizumab + NCRT	R0 100.0% pCR 42.8% MPR 66.6%
ESONICT‐2^160^	II	ChiCTR2100052784	Resectable	ESCC	Neoadjuvant	20	Toripalimab + NCT	ORR 70.0% pCR 16.7% MPR 41.7%
NEOCRTEC1901^161^	II	NCT04006041	Resectable	ESCC	Neoadjuvant	44	Toripalimab + NCRT	pCR 50% R0 98%
NEOCRTEC5010^162^	II	NCT04177797	Resectable	ESCC	Neoadjuvant	20	Toripalimab + NCRT	R0 87.5% TRAEs 100.0% ≥3 level TRAEs 100.0% pCR 18.8% MPR 43.8%
ESCORT‐NEO/NCCES01^163^	III	ChiCTR2000040034	Resectable	ESCC	Neoadjuvant	391	Camrelizumab + nab‐TP vs. Camrelizumab + TP vs. TP	pCR 28.0 vs. 15.4 vs. 4.7% MPR 59.1 vs. 36.2 vs. 20.9% R0 99.1 vs. 95.7 vs. 92.2%
CheckMate 648^70^	III	NCT03143153	Resectable	ESCC	Neoadjuvant	970	Nivolumab + CT vs. Nivolumab + Ipilimumab vs. CT	mOS 13.2 vs. 12.7 vs. 10.7 months ≥3 level TRAEs 47 vs. 32 vs. 36%
NIC‐ESCC2019^164^	II	NCT04225364	Resected in late stage	ESCC	Neoadjuvant	56	Camrelizumab + NRT	CPR 35.3% MPR 23.5%
/	II	NCT05918419	Resected in late stage	E/GEJ	Neoadjuvant	15	Serplulimab + NCRT	pCR 11.1% MPR 22.2%
NICE	II	NCT04744649	Local progressive stage	E/GEJ	Neoadjuvant	15	Triplimab + NCT	R0 100% MPR 92.9% pCR 78.6%

*Data sources*: Clinical registration website (www.clinicaltrials.gov) (www.chictr.org.cn), ASCO meeting (www.asco.org), ESMO meeting (www.esmo.org).

Abbreviations: *N*, the number of participants; ESCC, esophageal squamous cell carcinoma; EAC, esophageal adenocarcinoma; EC, esophageal cancer; CT, chemotherapy; CR, complete response; CRT, chemoradiotherapy; DCR, disease control rate; GEJ, gastroesophageal junction; mOS, median overall survival; mPFS, median progression‐free survival; ORR, objective response rate; pCR, pathological complete response; PR, partial response; R0, complete surgical resection; SD, stable disease; TRAEs, treatment‐related adverse events; MPR, major pathological response.

**TABLE 4:** A summary of clinical studies on perioperative immunotherapies for esophageal cancer.

Trial	Phase	Number	Stage	Type	Line	*N*	Intervention	Result
DANTE^165^	IIb	NCT03421288	Resectable	E/GEJ	Perioperative	295	Atezolizumab + NCT + surgery + Atezolizumab vs. surgery + CT	R0 92 vs. 91% MPR 24 vs. 15%
/	II	NCT03488667	Resectable	E/GEJ	Perioperative	26	Pembrolizumab + NCT + surgery + Pembrolizumab+ CT	R0 100% mDFS 25.7 months mOS 25.7 months pCR 19% ≥3 TRAEs 21/37
PERSIST	II	NCT04982939	Local advanced	E/GEJ	Perioperative	52	Sintilimab + NCT + surgery + Sintilimab vs. NCT + surgery + Sintilimab	pCR 26.9 vs. 4,8% MPR 69.2 vs. 28.6% Down‐staging 76.9 vs. 52.4%
GERCOR NEONIPIGA^166^	II	NCT04006262	Local advanced	E/GEJ	Perioperative	32	Nivolumab + Ipilimumab + surgery + Nivolumab	R0 100% pCR 58.6%
SHARED^167^	II	ChiCTR1900024428	Local advanced	E/GEJ	Perioperative	34	Sintilimab + NCRT + surgery + Sintilimab + CT	R0 100% pCR 38.2%% MPR 79.4% mDFS 17.0 months mEFS 21.1 months ≥3 TRAEs 50%
RAMSES/FLOT7^168^	II/III	NCT02661971	Resectable	E/GEJ	Perioperative	152	NCT + surgery + CT vs. Ramucirumab + NCT + surgery + CT	R0 82 vs. 96% DFS 21 vs. 32 months mOS 45 vs. 46 months MPR 26 vs. 29% ≥3 TRAEs 76 vs. 92%
ECOG‐ACRIN EA2174	II/III	NCT03604991	Local advanced	E/GEJ	Perioperative	278	Nivolumab + NCRT vs. Nivolumab + NCRT + surgery + Nivolumab + Ipilimumab	pCR 21.0 vs. 24.8% TRAEs 7.2 vs. 8.8%
CheckMate 577^169^	III	NCT02743494	Local advanced	E/GEJ	Adjuvant	794	Nivolumab vs. placebo	mDFS 22.4 vs. 11.0 months DMFS 28.3 vs. 17.6 months TRAEs 96 vs. 93%

*Data sources*: Clinical registration website (www.clinicaltrials.gov) (www.chictr.org.cn), ASCO meeting (www.asco.org), ESMO meeting (www.esmo.org).

Abbreviations: *N*, the number of participants; ESCC, esophageal squamous cell carcinoma; EC, esophageal cancer; CT, chemotherapy; CR, complete response; CRT, chemoradiotherapy; DCR, disease control rate; MDFS, distant metastasis‐free survival; GEJ, gastroesophageal junction; mOS, median overall survival; mPFS, median progression‐free survival; ORR, objective response rate; pCR, pathological complete response; PR, partial response; R0, complete surgical resection; SD, stable disease; TRAEs, treatment‐related adverse events; MPR, major pathological response.

**TABLE 5:** A summary of clinical studies on advanced first‐line immunotherapies for esophageal cancer.

Trial	Phase	Number	Stage	Type	Line	*N*	Intervention	Result
COMPASSION‐03^170^	Ib/II	NCT03852251	Advanced	G/GEJ	First line	96	Cardonilizumab + CT	ORR 65.9% DCR 92.0%
/	Ib/II	NCT04276493	Advanced	E/GEJ	First line	33	Zanidatamab vs. Tislelizumab + CT	ORR 72.7% mPFS 10.9 months ≥3 TRAEs 72.7% CR 5.3 vs. 0% PR 68.4 vs. 71.4% SD 26.3 vs. 28.6% PD 0 vs. 0%
AIO INTEGA^171^	II	NCT03409848	Advanced	E/GEJ	First line	97	Nivolumab + Trastuzumab + Ipilimumab vs. Nivolumab + Trastuzumab + CT	mOS 23.2 vs. 21.8months mPFS 3.2 vs. 10.7 months 1‐year OS 57 vs. 70%
/	II	NCT04821765	Advanced (oligofrecrudescence)	ESCC	First line	44	Tislelizumab + CRT	1‐year OS 83.2%1‐year PFS 83.2% ORR 84.1%
KSCC1902	II	jRCTs071190025	Advanced	E/GEJ	First line	42	Ramucirumab + CT	mPFS 4.0 months mOS 10.9 months RR 17.5% TTF 0.6 months DOR 14.3 months
AIO‐STO‐0218^172^	II	NCT03966118	Advanced	GEAC	First line	59	Ramucirumab + Avelumab + CT	mPFS 5.4 months mOS 10.6 months ORR 30.5% DOR 8.2 months CR 3.4 % PR 27.1 %SD 49.2 %PD 20.3 % DCR (CR+PR+SD) 79.7 %
/	II	NCT03647969	Advanced	G/GEJ	First line	120	CT VS. CT + Nivolumab + Ipilimumab	≥3 TRAEs 86 vs. 60% SAE 78 vs. 50% mPFS 5.7 vs. 6.6 months mOS 10 vs. 12 months ORR 45 vs. 48%
/	II	/	Advanced	EC	First line	49	Pembrolizumab	ORR 8% mOS 5.8 months
KCSG HN18‐17^173^	II	NCT03785496	Advanced	ESCC	First line	44	Spartalizumab	ORR 20.5% mPFS 3.2 months mOS 11.2 months DOR 24.7 months
ESCORT‐1st^174^	III	NCT03691090	Advanced	ESCC	First line	596	Camrelizumab + CT vs. placebo + CT	mOS 15.6 vs. 12.6 months mPFS 7.6 vs. 5.8 months 2‐year OS 35.9 vs. 23.8% 3‐year OS 25.6 vs. 12.8% ORR 72.1 vs. 62.1% ≥3 TRAEs 63.4 vs. 67.7%
LEAP‐015	III	NCT04662710	Advanced	G/GEJ	First line	15	Lenvatinib + Pembrolizumab + CT	ORR 73% DCR 93%
CheckMate648^70^	III	NCT03143153	Advanced	ESCC	First line	970	Nivolumab + CT vs. Nivolumab + Ipilimumab vs. CT	ORR 47 vs. 28 vs. 27% DOR 39 vs. 48 vs. 23% mOS 15.4 vs. 13.7 vs. 9.1 months mPFS 13.2 vs. 12.7 vs. 10.7 months
KEYNOTE‐590^175^	III	NCT03189719	Advanced	ESCC	First line	749	Pembrolizumab + CT vs. placebo + CT	mOS 12.3 vs. 9.8 months mPFS 6.3 vs. 5.8 months
JUPITER‐06^176^	III	NCT03829969	Advanced	ESCC	First line	809	Toripalimab + CT vs. placebo + CT	mOS 17.0 vs. 11.0 months mPFS 7.0 vs. 5.6 months ORR 69.3 vs. 52.1% DCR 89.1 vs. 82.1% mDoR 5.6 vs. 4.2 months ≥3 TRAEs 73.2 vs. 70.0%
ORIENT‐15^177^	III	NCT03748134	Advanced	ESCC	First line	659	Sintilimab + CT vs. placebo + CT	mOS 17.2 vs. 13.6 months mPFS 8.3 vs. 6.4 months mDoR 9.7 vs. 6.9 months ≥3 TRAEs 98.2 vs. 98.2%
RATIONAL‐306^146^	III	NCT03783442	Advanced	ESCC	First line	649	Tislelizumab + CT vs. placebo + CT	mOS 16.6 vs. 10.0 months mPFS 7.3 vs. 5.6 months ORR 63.5 vs. 42.4% mDoR 7.1 vs. 5.7 months TRAEs 96.6 vs. 96.3%

*Data sources*: Clinical registration website (www.clinicaltrials.gov) (www.chictr.org.cn), ASCO meeting (www.asco.org), ESMO meeting (www.esmo.org).

Abbreviations: *N*, the number of participants; ESCC, esophageal squamous cell carcinoma; EC, esophageal cancer; CT, chemotherapy; CR, complete response; CRT, chemoradiotherapy; DCR, disease control rate; DOR, duration of response; GEJ, gastroesophageal junction; mDOR, median duration of response; mOS, median overall survival; mPFS, median progression‐free survival; ORR, objective response rate; pCR, pathological complete response; PR, partial response; R0, complete surgical resection; SD, stable disease; TRAEs, treatment‐related adverse events; MPR, major pathological response.

**TABLE 6:** A summary of clinical studies on advanced non‐first‐line immunotherapies for esophageal cancer.

Trial	Phase	Number	Stage	Type	Line	*N*	Intervention	Result
/	II	/	Advanced	ESCC	Second line	41	Camrelizumab + Nimotuzumab	ORR 36% mOS 12.62 months mPFS 9.17 months DCR 81% TRAEs 60%
PRODIGE 59‐DURIGAST^178^	II	NCT03959293	Advanced	G/GEJC	Second line or above	96	Durvalumab + CT vs. Durvalumab + Tremelimumab + CT	4‐month PFS 44.7 vs. 57.8% mPFS 3.8 vs. 5.9 months DCR 68.9 vs. 73.8% mOS NE vs. 10.1 months ≥3 TRAEs 50.0 vs.47.8%
ATTRACTION‐1^179^	II	/	Advanced	ESCC	Second line	65	Nivolumab	3‐year OS 10.9% 5‐year OS 6.3%
RAMONA^180^	II	NCT03416244	Advanced	ESCC	Second line or above	69	Nivolumab vs. Nivolumab + Ipilimumab	≥3 TRAEs 100 vs. 9.52%
AdvanTIG‐203	II	NCT04732494	Advanced	ESCC	Second line or above	280	Ociperlimab vs. placebo	ORR 30.6 vs. 20.6%
ESCORT^181^	III	NCT03099382	Advanced	ESCC	Second line or above	448	Camrelizumab vs. CT	mOS 8.3 vs. 6.2 months mPFS 1.9 vs. 1.9 months mDoR 7.4 vs. 3.4 months ≥3 TRAEs 19.0 vs. 40.0%
ATTRACTION‐3^182^	III	NCT02569242	Advanced	ESCC	Second line or above	419	Nivolumab vs. CT	mOS 10.9 vs. 8.4 months mPFS 1.7 vs. 3.4 months ORR 19.3 vs. 21.5% mDoR 6.9 vs. 3.9 months
KEYNOTE‐181^183^	III	NCT02564263	Advanced	ESCC/EAC	Second line or above	387	Pembrolizumab vs. CT	mOS 7.1 vs. 7.1 months 12‐month OS 43 vs. 20% TRAEs 95.5 vs. 97.3%
RATIONALE 302^184^	III	NCT03430843	Advanced	ESCC	Second line or above	512	Tislelizumab vs. CT	mOS 8.6 vs. 6.3 months mPFS 1.6 vs. 2.1 months mDoR 7.1 vs. 4.0 months ORR 20.3 vs. 9.8% ≥3 TRAEs 46 vs. 68%
Advanced and first line or second line or above								
ESCORT‐RWS	foresight	NCT04616040	Advanced	ESCC/EAC	First line or second line or above	624	Contains at least Camrelizumab (first line vs. second line vs. third line or above)	CR 4.0 vs. 2.3 vs. 3.5% PR 50.2 vs. 29.1 vs. 24.6% SD 41.9 vs. 48.6 vs. 40.4% PD 2.6 vs. 19.4 vs. 31.6% ORR 54.2 vs. 31.4 vs. 28.1% mPFS 10.1 vs. 7.9 vs. 7.9 months mOS 17.5 vs. 14.0 vs. 12.8 months TRAEs 88.9% ≥3 TRAEs 40.9%
/	Ib	jRCT2080224975	Advanced	ESCC/EAC	First line or second line or above	107	Futibatinib + Pembrolizumab (first line) vs. Futibatinib + Pembrolizumab (second line or above) vs. Futibatinib + Pembrolizumab + CT (first line)	cPR 40 vs. 6.1 vs. 68.42% CR 2.9 vs. 0 vs. 0% ORR 42.9 vs. 6.1 vs. 68.4% DCR 71.4 vs. 51.0 vs. 89.5% mDOR 16.0 vs. 4.7 vs. 5.6 months
/	II	NCT03766178	Advanced	ESCC	First line or second line or above	42	Camrelizumab + Nimotuzumab	ORR 36% mOS 12.62 months mPFS 9.89 months DCR 81%

*Data sources*: Clinical registration website (www.clinicaltrials.gov) (www.chictr.org.cn), ASCO meeting (www.asco.org), ESMO meeting (www.esmo.org).

Abbreviations: *N*, the number of participants; ESCC, esophageal squamous cell carcinoma; EC, esophageal cancer; CT, chemotherapy; CR, complete response; CRT, chemoradiotherapy; DCR, disease control rate; DOR, duration of response; GEJ, gastroesophageal junction; mDOR, median duration of response; mOS, median overall survival; mPFS, median progression‐free survival; ORR, objective response rate; pCR, pathological complete response; PR, partial response; R0, complete surgical resection; SD, stable disease; TRAEs, treatment‐related adverse events; MPR, major pathological response.

**TABLE 7:** A summary of clinical studies on ongoing immunotherapies for esophageal cancer.

Trial	Phase	Number	Stage	Type	Line	*N*	Intervention	Primary endpoint	Main result
HangzhouCH09	II	NCT03200691	Resectable	ESCC	Neoadjuvant	21	Camrelizumab + RT	2‐year OS	Recruiting
/	II	NCT06385730	Resectable	ESCC	Neoadjuvant	60	Triplimab vs. Triplimab + NRT	MPR	Recruiting
POINTS	II	ChiCTR2300073898	Resectable	ESCC	Neoadjuvant	30	Camrelizumab + NCT	pCR	Recruiting
/	IIa	ChiCTR2300075075	Resectable	ESCC	Neoadjuvant	54	Toripalimab + NCT	pCR	Recruiting
NICCE	/	NCT05028231	Resectable	ESSC	Neoadjuvant	46	PD‐1/PD‐L1 antibody + NCT	pCR	Recruiting
KEYSTONE002^185^	III	NCT04807673	Resectable	ESCC	Neoadjuvant	342	Pembrolizumab + NCT vs. NCT	EFS	Recruiting
/^186^	II	NCT05176002	Resectable	ESCC	Neoadjuvant	26	Camrelizumab + NRT	MPR, AE	Recruiting
iCROSS	II/III	NCT04973306	Resectable	ESCC	Neoadjuvant	176	Tislelizumab + NCRT vs. NCRT	pCR, OS	Recruiting
NICE‐RT	II	NCT05650216	Resectable	ESCC	Neoadjuvant	50	Camrelizumab + NCRT	Safety, pCR	Not yet recruiting
/	II	NCT04229459	Resectable	ESCC	Neoadjuvant	31	Cetuximab + Nivolumab + NCRT	pCR, PFS, Safety	Recruiting
/	II	NCT05323890	Resectable	ESCC	Neoadjuvant	15	Tislelizumab + NCRT	pCR, PCR	Recruiting
TINES	IV	NCT05603065	Resectable	ESCC	Neoadjuvant	32	Tislelizumab + NCT vs. Tislelizumab + NRT	pCR	Active, not recruiting
/	Ib/II	NCT04460066	Resectable	ESCC	Neoadjuvant	70	PD‐L1‐antibody ZKAB001 + NCT	MPR	Active, not recruiting
/	III	NCT04821843	Resectable	ESCC	Neoadjuvant	2000	Nimotuzumab + NCT vs. nimotuzumab + NCRT vs. NCT vs. NCRT	OS	Recruiting
PPIO‐004‐EC001	II	NCT05880082	Resectable	ESCC	Neoadjuvant	62	Tislelizumab + NCT	OR, MPR	Not yet recruiting
NICE‐2^187^	II	NCT05043688	Resectable	ESCC	Neoadjuvant	204	SHR‐1210+NCT vs. SHR‐1210+NRT vs. NRT	pCR	Not yet recruiting
NATION‐2203^188^	II/III	NCT05213312	Resectable	ESCC	Neoadjuvant	90	Nivolumab + NCT vs. NCT	pCR	Recruiting
/	III	NCT05244798	Resectable	ESCC	Neoadjuvant	420	Sintilimab + NCT vs. NCT	pCR	Not yet recruiting
/	Ib/II	NCT05541445	Resectable	ESCC	Neoadjuvant	40	Pembrolizumab + NCT	MPR	Recruiting
/	Ib/II	NCT05743504	Resectable	ESCC	Neoadjuvant	32	Tiragolumab + Atezolizumab + NCT	pCR	Not yet recruiting
/^189^	II	NCT05821452	Resectable	ESCC	Neoadjuvant	40	Camrelizumab + NCT vs. NCT	R0, MPR	Not yet recruiting
PALACE‐2^190^	II	NCT04435197	Resectable	ESCC	Neoadjuvant	143	Pembrolizumab + NCRT	pCR	Recruiting
/	II	NCT06056336	Resectable	ESCC	Neoadjuvant	73	Tislelizumab + NCT	2‐year DFS in non‐pCR patients	Recruiting
CRISEC^159^	II	NCT04776590	Resectable	ESCC	Neoadjuvant	30	Tislelizumab + NRT	pCR	Recruiting
RARE	II	NCT05941481	Resectable	EC	Neoadjuvant	21	Tislelizumab + NCRT	pCR	Active, not recruiting
/	II	NCT05807542	Resectable	ESCC	Neoadjuvant	20	Tislelizumab + NCT	pCR	Recruiting
/	II/III	NCT03604991	Resectable	E/GEJ	Neoadjuvant	278	NCRT vs. Nivolumab + NCRT vs. Nivolumab vs. Nivolumab + Ipilimumab	pCR, DFS, OS, AEs	Suspended
/	II	NCT06121700	Local advanced	G/GEJC	Neoadjuvant	55	ICIs + NCRT	OS	Recruiting
/	I/II	NCT05394415	Local advanced	ESCC	First line	30	Tislelizumab + CRT	Safety	Recruiting
STARS	II	NCT06422858	Local advanced	ESCC	First line	37	Serplulimab + CRT	1‐year PFS	Recruiting
/	II	ChiCTR2300071203	Oligometastatic	EC	First line	50	Sintilimab + CRT	PFS	Recruiting
FUTURE‐2	II	NCT06401447	Local advanced	ESCC	First line	50	Sintilimab + CRT	PFS	Recruiting
KEYNOTE‐975^191^	III	NCT04210115	Local advanced	G/GEJC	First line	703	Pembrolizumab + CRT vs. placebo + CRT	EFS, OS	Active, not recruiting
MK‐3475‐06B	I/II	NCT05319730	Advanced	ESCC	Second line or above	200	Pembrolizumab + MK‐4830 + CT vs. CT	DLT. AEs, ORR	Recruiting
/	II	NCT05265962	Advanced	ESCC	Second line	85	Penpulimab + CT	OS	Not yet recruiting
/	II	NCT05942573	Advanced	G/GEJC	Second line	107	Serplulimab + CT	6‐month PFS	Recruiting
Escape	III	NCT05737563	Advanced	ESCC	Second line	380	Teripulimab/Carrelizumab + CT vs. CT	OS	Recruiting
RATIONALE‐311^192^	III	NCT03957590	Local advanced	ESCC	First line	370	Tislelizumab + CRT vs. placebo + CRT	PFS	Active, not recruiting

*Data sources*: Clinical registration website (www.clinicaltrials.gov) (www.chictr.org.cn).

Abbreviations: *N*, the number of participants; CRT, chemoradiotherapy; CT, chemotherapy; DCR, disease control rate; DLT, dose limiting toxicity; ESCC, esophageal squamous cell carcinoma; EC, esophageal cancer; GEJ, gastroesophageal junction; ORR, objective response rate; OS, median overall survival; pCR, pathological complete response; PFS, median progression‐free survival; TRAEs, treatment‐related adverse events.

However, despite the promising prospects displayed by ICIs in treating EC, many questions still require further research. For example, which ICI is most cost effective in EC?^193^ What are its long‐term effects? Optimal timing for usage and patient selection criteria are also areas that require further elucidation through more extensive and larger‐scale clinical trials.

### 3.3 Combination therapies

**FIGURE 1:**
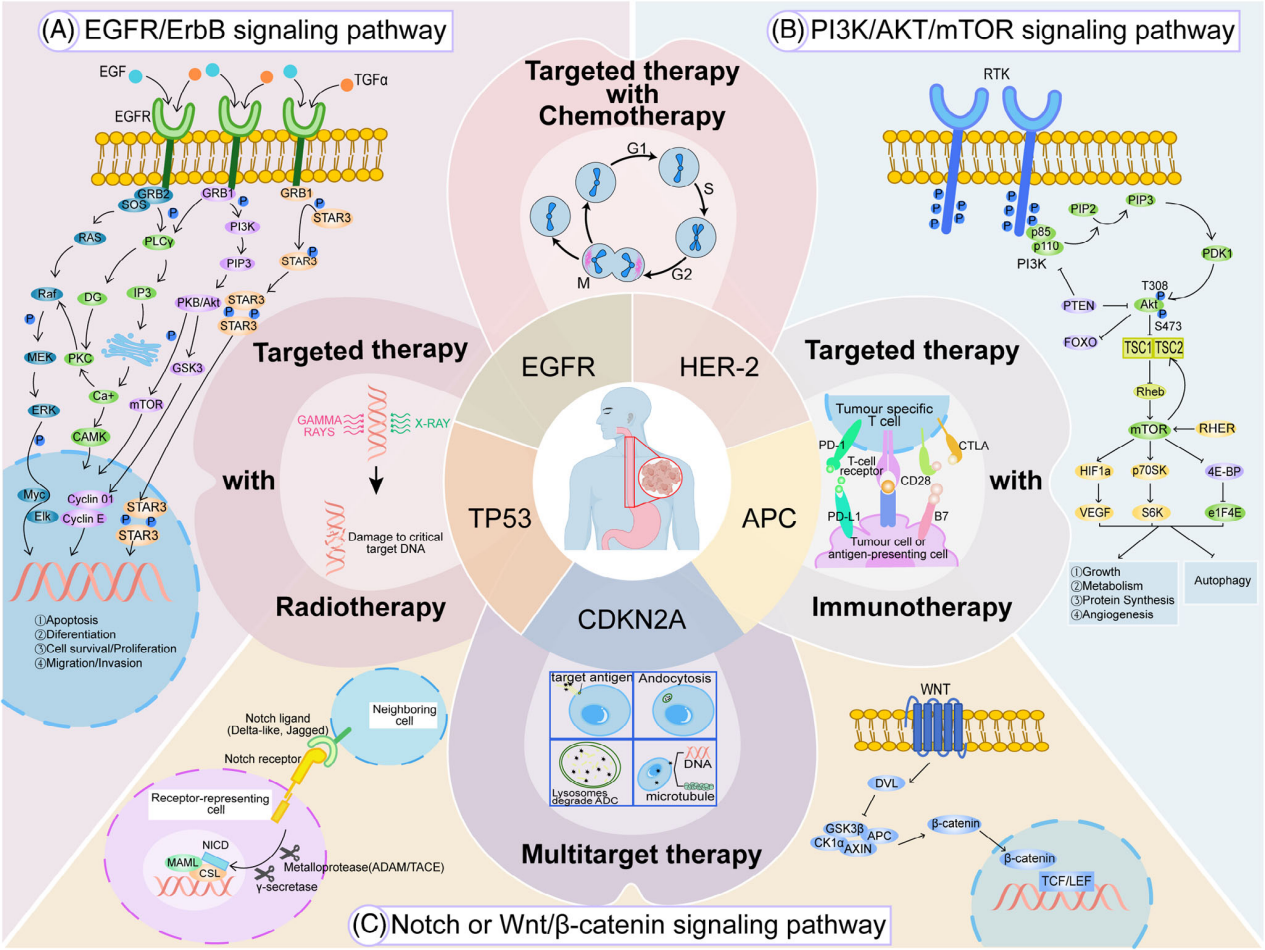
Potential strategies and potential targets for treating EC. Current the EC's research focuses on pivotal genes EGFR, HER2, TP53, CDKN2A, and APC, targeting key pathways in cell regulation and signaling. We highlight four novel therapeutic strategies: combined targeted therapy with chemotherapy, radiotherapy, immunotherapy, and multitarget approaches. Mechanistic insights into esophageal carcinogenesis underscore alterations in EGFR/ErbB, PI3K/AKT/mTOR, and Notch/Wnt/β‐catenin pathways, driving proliferative and survival signaling cascades. CDKN2A, cyclin‐dependent kinase inhibitor 2A; EC, esophageal cancer; EGFR, endothelial growth factor receptor; HER2, human epidermal growth factor receptor 2; mTOR, mammalian target of rapamycin; PI3K, phosphatidylinositol 3‐kinase; TP53, tumor protein 53.

The success of targeted therapy often depends on the tumor cells’ sensitivity to target inhibition. As the TME changes, tumor cells may develop resistance through various mechanisms. Therefore, relying solely on the inhibition of a single target often do not result in long‐term, sustained efficacy. This makes combination therapy crucial (Figure 1).

#### 3.3.1 Targeted therapy combined with CT

Targeted therapy specifically aims at molecular targets present in tumor cells, whereas CT targets rapidly dividing cells by disrupting their cell cycle. Combining the two can reduce the development of resistance to targeted therapy and utilizes different mechanisms of action. Furthermore, through proper dosage design, the addition of targeted therapy can influence the dosage of CT, thereby reducing the toxic side effects of CT and improving patient tolerance. In the Phase III ToGA clinical study, the Trastuzumab and CT group had an mOS of 13.8 months compared with 11.1 months for the CT‐alone group,^194^ establishing it as a standard treatment for HER2‐positive GEJ cancer. In addition, in a Phase III study, the combination of Gefitinib and CT as a second‐line treatment for metastatic EC provided palliative benefits for patients with short expected survival, although it did not improve OS.^195^ Furthermore, clinical investigations involving the combination of Cetuximab or Bevacizumab with CT in advanced EC patients did not yield benefits in terms of OS and PFS.^196^ Therefore, the current evidence from evidence‐based medicine on the combination of targeted therapy and CT remains very limited.

#### 3.3.2 Targeted therapy combined with radiotherapy

Some scholars have suggested that targeted therapy can synergize with radiotherapy by enhancing the sensitivity of EC to radiation, but current research data on the combined use of targeted therapy and radiotherapy remain very limited. There is a lack of clinical studies for advanced EC, and a combination model of radiotherapy with targeted therapy has not yet been established. Targeted agents against VEGF and VEGFR have the potential to remodel tumor vasculature, which can decrease the population of hypoxic cells within tumors and subsequently enhance the efficacy of radiotherapy. Thus, how to use antiangiogenic drugs in combination with radiotherapy to achieve minimal adverse reactions presents significant value and challenges. Current studies have not shown that concurrent targeted therapy with radiotherapy significantly increases or adds extra toxicity.^197^ Preclinical studies have shown that radiotherapy can regulate the expression of PD‐L1 on the surface of tumor cells and immune cells in the TME, as well as increasing the infiltration of T cells in the TME.^198^ A meta‐analysis comparing the outcomes of combined targeted/immunotherapy with chemoradiotherapy alone in patients with locally advanced EC revealed that the addition of targeted or immunotherapeutic agents significantly improved PFS, pathological remission rates, and ORR. Importantly, this combination did not elevate the risk of severe adverse events, suggesting it as a safe and effective addition to standard chemoradiotherapy protocols.^199^

#### 3.3.3 Targeted therapy combined with immunotherapy

As the majority of solid tumors often overexpress angiogenic factors, the addition of antiangiogenic therapy can to some extent normalize the aberrant tumor vasculature, enhance the infiltration of immune effector cells into the tumor, and thereby reverse the immunosuppressive microenvironment to one that promotes immunity. Recent studies have highlighted the efficacy and manageable toxicity of combining Anlotinib with TQB2450, a novel PD‐L1 inhibitor, as a first‐line treatment for advanced ESCC. This combination achieved an ORR of 69.6%, a disease control rate (DCR) of 93.5%, and a mPFS of 9.92 months. Additionally, at the 2024 American Society of Clinical Oncology (ASCO) GI conference, a study reported that combining immunotherapy with Apatinib as a second‐line treatment for EC patients progressing on prior immune therapy showed an ORR of 36.8%, an mPFS of 4.6 months, and an mOS of 7.5 months. Synergistic antitumor activity characteristics were also validated in the CP‐MGAH22‐05 single‐arm study combining a novel HER2 monoclonal antibody with Pembrolizumab.^200^ Ongoing trials, like LEAP‐014 studying Parbociclib, Lenvatinib, and CT, and ANSWER examining Panitumumab with CT, with or without Anlotinib, are garnering significant attention for their promising efficacy. In the KEYNOTE‐811 randomized controlled trial involving the combination of HER‐2 antibodies, the combination of Pembrolizumab and Trastuzumab plus CT significantly increased the PFS of patients with metastatic HER2‐positive GEJ (10.0 m vs. 8.1 m, *p* = 0.0002),^131^ but a similar regimen did not meet the primary endpoint in the JACOB study.^201^

#### 3.3.4 Multitarget therapy

Single‐target therapies often face significant challenges, as tumor cells can adapt through various molecular and cellular mechanisms to evade the effects of targeted drugs. This adaptation can lead to the development of more aggressive and metastatic tumor phenotypes, while compensatory pathways may support tumor cell survival and progression.^202^ Combination therapy can improve the deficiencies of single‐target treatment, exert synergistic anticancer effects, overcome clonal heterogeneity, and reduce the probability of resistance. However, this also implies to some extent the accumulation of side effects from combination therapy, where drug interactions or superpositions can lead to a certain probability of side effects.^203^ Therefore, BsAb drugs (bispecific antibodies),^204^ ADC drugs,^205^ and MTDL drugs (multitarget‐directed ligands)^206^ have emerged. BsAbs, engineered through cell fusion or recombinant DNA technology, can simultaneously bind two distinct antigens or different epitopes of the same antigen, enhancing therapeutic effects. ADCs link antigen‐targeting antibodies with cytotoxic drugs, enabling precise delivery of the cytotoxic agent to tumor cells. Meanwhile, MTDLs utilize tumor‐specific probes to identify and target multiple pathways simultaneously. These ligands are discovered through random screening or framework combination methods, resulting in novel hybrid molecules that exhibit multifunctional activity against various targets. In the AK104‐IIT‐014 study, the BsAb Cadonilimab, targeting PD‐1 and CTLA‐4, showed an impressive ORR of 86.7% in EC patients. Similarly, in a Phase II clinical trial, the ADC drug 9MW2821, which targets Nectin‐4, achieved an ORR of 30% and a DCR of 73.3% in patients with advanced EC, with ongoing enrollment and evaluation. Additionally, other BsAbs like Bintrafusp alfa (targeting PD‐L1 and TGF‐β) and MTDLs such as DP303c (targeting FGFR, CSF1R, and KDR) and KC1036 (targeting AXL, VEGFR2, and FLT3) are in Phases I and II clinical trials for EC. These studies aim to provide new therapeutic options and hope for improved clinical outcomes.

## 4 CHALLENGES AND FUTURE DIRECTIONS

### 4.1 Overcoming drug resistance

Despite significant advancements in targeted therapeutic agents, resistance remains a pervasive challenge, often emerging due to genetic mutations, activation of alternative signaling pathways, or alterations in TME. In EC, primary mechanisms of resistance to HER2‐targeted therapies include mutations and deletions in the HER2 gene. As HER2‐targeted treatments advance, EC cells may evade therapeutic effects through genetic mutations or by downregulating HER2 expression.^207^ Additionally, the amplification of the ERBB2 gene is a prevalent resistance mechanism, leading to abnormal activation of the HER2 signaling pathway and conferring resistance to EGFR TKIs.^208^ Continuous cell proliferation and division in cells with ERBB2 amplifications can further enhance their resistance capabilities. Moreover, EC cells can upregulate the expression of drug efflux pumps, such as multidrug resistance proteins and other ATP‐binding cassette transporters. This results in a reduced effective concentration of the drugs within the cells.^209^ Activation of the HER2 receptor typically engages the PI3K/AKT and MAPK signaling pathways, significantly contributing significantly to drug resistance.^207^ Concurrently, the TME undergoes gradual modifications during treatment, enabling EC cells to adapt to the in vivo environment and resist therapeutic interventions.^209^ Consequently, drug resistance poses a major obstacle in the efficacy of targeted therapies. To address these challenges, combination therapies involving radiotherapy and immunotherapy are being explored. These combinations aim to disrupt the survival mechanisms of EC cells through multiple pathways, thereby mitigating the development of drug resistance. In clinical practice, it has also been found that chemotherapeutic can alter the sensitivity of patients to targeted drugs, which might be related to the synergistic effects of targeted therapy and CT,^210^ although the specific mechanisms are unclear. To improve the efficacy of targeted drugs, multitargeted therapies have been developed. For example, Afatinib can covalently bind to the kinase domains of EGFR (ErbB1), HER2 (ErbB2), and HER4 (ErbB4), irreversibly inhibiting tyrosine kinase autophosphorylation, leading to the downregulation of ErbB signaling. Research is focused on identifying synergistic anticancer drug combinations by examining the impact of risk‐associated mRNA and miRNA on dysfunctional pathways and pathway interactions.^211^ The Graph Convolutional Network model also emerges as a promising approach in this context.^212^ Additionally, optimizing the dosages and administration methods of each drug within combination therapies presents another viable avenue for exploration. Of course, the optimal and most effective strategy would involve tailoring combination treatment plans based on patients' genomic profiles and the characteristics of their TME, thereby identifying the root causes of resistance and devising personalized therapeutic regimens. In vitro studies have demonstrated that low NTRK2 expression can confer resistance to Afatinib through the activation of the MAPK/ERK signaling pathway, regardless of EGFR expression levels.^213^ Therefore, utilizing multitarget drugs, combining them with downstream pathway inhibitors, and targeting different molecular entities are crucial strategies to overcome drug resistance in targeted therapy for EC.

### 4.2 Biomarker discovery and patient selection

According to ASCO guidelines, patients with EC should undergo comprehensive biomarker assessments, including HER2, PD‐L1, dMMR/MSI‐H, NTRK, BRAF, RET, and tumor mutational burden (TMB) testing. These biomarkers are essential for guiding precision medicine approaches, with particular emphasis on HER2 gene testing in cases of esophageal and esophagogastric junction adenocarcinoma. Numerous studies have demonstrated that tumors exhibiting positive PD‐L1 expression, dMMR/MSI‐H, or elevated TMB are more responsive to immunotherapy. Consequently, irrespective of HER2 status, the presence of PD‐L1 protein expression detected through immunohistochemistry can justify the initiation of immunotherapy‐based combination treatment regimens as first‐line therapies. Beyond PD‐L1, guidelines endorse using pembrolizumab and dostarlimab for dMMR/MSI‐H status, and Pembrolizumab for high TMB (TMB‐H) patients.

To further clarify the pathogenesis and molecular typing of ESCC and search for molecular biomarkers, Liu et al.^6^ conducted whole‐genome sequencing on 508 pairs of ESCC tissue samples and protein and phosphoproteome sequencing on 94 samples, discovering heterogeneity in CDK4/6 and NRF2 gene expression among different ESCCs. Further examination of gene expression profiles from 84 ESCC tumors identified that the chemokine CCL18 promotes tumor cell proliferation through the JAK2/STAT3 signaling pathway.^214^ Additionally, research has shown that mutations in the NFE2L2 gene can disrupt its tumor‐suppressive functions, correlating with poorer prognoses.^215^ Single‐cell transcriptomic analyses comparing normal esophageal tissues with ESCC tissues have uncovered the presence of immune checkpoint genes such as LAG3 and HAVCR2, which may play pivotal roles in shaping the TME and contributing to ESCC heterogeneity.^216^ However, a prospective clinical translational study indicated that while TMB and clonal TMB are indicators of tumor immunogenicity and potential predictors of immunotherapy efficacy, they do not reliably forecast the effectiveness of CT combined with PD‐1 antibody treatments in ESCC. Notably, only copy number variation‐corrected TMB (cc‐TMB) emerged as a dependable biomarker for predicting the success of the “PD‐1 antibody + CT” therapeutic strategy. Moreover, prognostic insights can be gained from analyzing TME elements, such as the CD8A/C1QA mRNA ratio,^217^ CD68 macrophage‐associated PD‐1H expression,^218^ and CD177 Tregs presence.^219^

From a clinical pathological standpoint, minimal residual disease analysis, which detects tumor DNA in circulating free DNA, effectively predicts complete pathological remission in ESCC patients.^220^ In terms of tumor prognosis, vascular invasion is closely related to the tumor's ability to metastasize; especially in EC, the degree of venous invasion is positively correlated with the rate of distant metastasis. This may be because tumor cells can migrate to other organs through the bloodstream, further promoting tumor development and metastasis.^221^ Currently, the TNM staging system includes vascular invasion as a significant prognostic factor, often reported alongside lymphatic invasion. However, these two forms of invasion may have distinct prognostic implications, with lymphatic invasion typically associated with a poorer prognosis.^222^ In the future, will vascular invasion be considered as a separate factor in the TNM staging of ESCC? Furthermore, CD8+ T cell infiltration in the TME is positively correlated with EC prognosis, and CD4+ T cell infiltration is also prognostically significant.^223^ This highlights the importance of considering the immune microenvironment alongside vascular invasion. For surgical patients, nutritional status is a critical prognostic factor. Parameters such as hemoglobin levels, body mass index, albumin (ALB), prognostic nutritional index,^224^ and inflammatory markers like the neutrophil‐to‐lymphocyte ratio and C‐reactive protein to ALB ratio^225^ influence patient outcomes. Studies have shown that skeletal muscle loss is significantly associated with poor surgical outcomes, hence, when assessing baseline characteristics, postoperative recovery, and long‐term prognosis of EC patients, postoperative changes in fat and skeletal muscle should also be monitored and considered.^226^

Despite the gradual discovery of numerous potential biomarkers, none have been recommended for clinical implementation due to a lack of validation studies. One of the primary limitations is the insufficient sample size available for such studies. Moreover, in clinical practice—particularly concerning Barrett's esophagus and EAC—the sensitivity and specificity of currently identified biomarkers have not yet met clinical standards. This shortfall is partly due to existing detection technologies being inadequate for identifying biomarkers at low concentrations. As a result, the majority of these biomarkers have not advanced to clinical practice. Additionally, the standards required for the clinical use of biomarkers have not been clearly defined or standardized.^227^ More importantly, the expression of the same biomarker can vary significantly among different patients. Factors such as individual genetic differences and tumor heterogeneity influence biomarker expression, leading to substantial variability.^228^ Consequently, the universal applicability of these biomarkers remains one of the critical challenges that must be addressed. In the future, efforts should be directed toward validating the efficacy and reliability of biomarkers by conducting multicenter, large‐scale clinical trials. Leveraging advanced technologies such as liquid biopsy, proteomics, and genomics, individualized biomarker screening strategies should be developed in conjunction with patients’ genetic backgrounds and tumor characteristics. Furthermore, standardized testing and validation protocols need to be established to promote the clinical application of these biomarkers.

### 4.3 Enhancing therapeutic efficacy

The current effectiveness of targeted therapy for EC remains unsatisfactory, with effectiveness rates between 15 and 30%. In addition to the aforementioned combined treatment strategies and multitarget synergistic approaches to overcoming resistance, the development of novel drug formulations is also crucial for enhancing the efficacy of traditional treatments.

ADC, which stands for antibody–drug conjugate, consists of an antibody, a highly potent toxic drug molecule, and a linker. ADCs are capable of specifically recognizing antigens and releasing payloads to kill target cells. The DESTINY‐Gastric02 trial exemplifies the potential of ADCs in EC treatment. In this study, Trastuzumab deruxtecan, an ADC targeting HER2, was administered to patients with inoperable HER2‐positive GEJ cancer. The results demonstrated an ORR of 38%, with 4% of patients achieving CR and 34% achieving PR. Additionally, sustained remission was observed in patients who had previously progressed after receiving Trastuzumab‐containing treatment regimens.^229^ This shows the promising clinical efficacy of ADCs, however, the drawback is the lack of data from Asian patients. Of course, numerous studies are gradually exploring ADC treatment research for EC, and it is believed that ADCs will bring a new chapter in the treatment of EC.

Furthermore, proteasome inhibitors are a type of multisubunit enzyme complex that can control cell proliferation, division, and apoptosis by degrading proteins that regulate the cell cycle and are involved in the apoptosis pathway. Carfilzomib, a second‐generation proteasome inhibitor, has shown significant antitumor activity against ESCC in both in vitro and in vivo studies. The antitumor effects of Carfilzomib are attributed to its ability to induce mitochondrial apoptosis and reprogram cellular metabolism within ESCC cells.^230^

### 4.4 Novel immune drug

TIGIT functions as an immune checkpoint by interacting with its ligand, CD155, which is expressed on both tumor cells and immune cells. This binding inhibits the activity of NK cells and T cells, thereby diminishing the antitumor immune response.^231^ In the Phase II KEYVIBE‐005 clinical trial,^103^ the combination of vibostolimab (an anti‐TIGIT antibody) with pembrolizumab and CT demonstrated enhanced efficacy compared with Pembrolizumab combined with CT alone. Specifically, the ORR improved from 45 to 53%, while PFS and OS were extended to 10.4 and 18.0 months, respectively. Similarly, the Phase III SKYSCRAPER‐08 study investigating Tiragolumab, another anti‐TIGIT antibody, revealed a significant increase in PFS by 0.8 months compared with the control group, highlighting the potential of TIGIT‐targeted therapies as a promising first‐line treatment for advanced EC.

LAG‐3 is another critical immune checkpoint that negatively regulates T cell activation and function.^232^ High expression levels of LAG‐3 in EC are significantly correlated with CTLA‐4 expression and are associated with poorer prognoses.^233^ Research indicates that simultaneous blockade of LAG‐3 and PD‐1 is more effective in restoring immune homeostasis and enhancing tumor immunity than targeting either checkpoint alone.^234^ This dual inhibition strategy offers a more robust approach to overcoming immune suppression within the TME. Chimeric antigen receptor T‐cell (CAR‐T) therapy represents a personalized treatment modality that involves genetically modifying T cells to recognize and target specific cancer antigens.^235^ Preclinical studies in EC have explored CAR‐T cells targeting various antigens, including MUC1, HER‐2,^236^ B7‐H3,^237^ and EphA2,^238^ among others. For instance, CAR‐T cells targeting CD276 have shown efficacy in eliminating ESCC, leading to significant tumor reduction and extended survival in murine models.^239^ Additionally, a Phase Ib/II clinical trial evaluating CT041 CAR‐T therapy for GEJ cancer reported an extended OS of 10.8 months, demonstrating the therapeutic potential of CAR‐T cells in EC. NK cells play a crucial role in the innate immune response by recognizing and destroying abnormal and tumor cells through interactions with cell adhesion molecules on their surface.^240^ Tumor cells often exhibit low levels of MHC‐I molecules, making them more susceptible to NK cell‐mediated cytotoxicity.^241^ However, they can evade NK cell activity by engaging inhibitory killer cell immunoglobulin‐like receptors through their MHC‐I molecules. CAR‐NK therapies aim to boost NK cell cytotoxicity, but challenges like limited persistence and the immunosuppressive TME remain.^242^ Dendritic cells (DCs) are crucial for antigen presentation and regulating immune responses by attracting naive and memory T cells.^243^ In a clinical trial with seven ESCC patients,^244^ SART1 peptide‐pulsed monocyte‐derived DCs enhanced immune responses, increasing interferon‐gamma levels. This approach underscores the potential of DC vaccines in boosting antitumor immunity and improving patient outcomes.

### 4.5 The translation from clinical trials to clinical practice

The number of clinical trials investigating targeted therapies for EC remains exceedingly limited. Moreover, the majority of existing trials primarily focus on evaluating the efficacy at various stages of treatment, and there is a lack of consistency in the clinical treatment standards for many targeted agents. The therapeutic effects of targeted drugs in real‐world practice are often less favorable than those reported in clinical trials. This discrepancy is closely related to patient heterogeneity, concomitant medications, and treatment adherence. Particularly in elderly patients and those with comorbidities, the adverse effects of targeted therapies may be more pronounced, and their safety profiles may fall short of what is demonstrated in clinical trials. During the transition from clinical trials to clinical practice, additional attention should be paid to individual patient biomarkers, genomic characteristics, and metabolic differences. The variability in drug responses among different patients is substantial and represents a challenge that is difficult to capture within the confines of clinical trials. Future research should prioritize developing personalized targeted therapies, as this will be key to improving the prognosis of EC patients.

## 5 CONCLUSION

Beginning with a detailed exploration of EC's molecular pathogenesis, the review meticulously unpacks the genetic and epigenetic landscapes that underpin this malignancy, emphasizing the pivotal roles played by key oncogenes, tumor suppressor genes, and the critical signaling pathways that orchestrate the cancer's behavior. Advances in molecular biology and pharmacotherapy have clarified the roles of genetic and epigenetic factors in EC onset and demonstrated the feasibility of targeting molecular pathways like EGFR, HER2, VEGFR, and immune checkpoints. This foundational understanding sets the stage for subsequent discussions, positioning molecular pathogenesis as the cornerstone of advancements in EC treatment strategies. After that, we review and critically analyze the latest clinical research progress of targeted and immunotherapy. It is presented a hopeful yet challenging outlook for targeted therapies, reaffirming that while targeted treatments like Anlotinib and Apatinib show promise in second‐line treatments, monoclonal antibodies like Trastuzumab have demonstrated efficacy in HER‐2 positive cases, but resistance issues and the need for biomarker‐driven patient selection remain key barriers. Integrating ICIs, such as Pembrolizumab, into treatment regimens marks a major paradigm shift, offering new hope for improving the prognosis of patients with early and advanced stages of the disease. The exploration of combination therapies proposes a synergistic approach integrating targeted therapy, immunotherapy, and TME modulation. This section outlines the potential of leveraging the interplay between various treatments to circumvent resistance mechanisms and achieve sustained therapeutic responses. Additionally, the review delves into the intricate dynamics of the TME and its role in cancer progression and therapy resistance. Understanding the TME's complexity is crucial for developing effective treatments to disrupt tumor cell immune evasion tactics.

Future efforts should focus on refining and expanding combinations of molecular targets, enhancing patient‐specific treatments, and overcoming resistance mechanisms through combination therapies and the discovery of new drugs. Moreover, a deeper understanding of the TME may lead to more effective immunotherapy strategies. The quest for the best treatment strategies for EC remains challenging, yet the constantly evolving scientific landscape fosters optimism for better and more effective treatments that hope to improve patient survival rates and quality of life.

## AUTHOR CONTRIBUTIONS

*Conceptualization, data curation, writing—original draft*: Wenjing Wang. *Conceptualization, data curation, validation, writing—review and editing*: Lisha Ye. *Conceptualization, validation, writing—review and editing*: Huihui Li. *Project administration, writing—review and editing*: Weimin Mao. *Project administration, supervision, writing—review and editing*: Xiaoling Xu.

All authors have read and approved the final manuscript.

## CONFLICT OF INTEREST STATEMENT

The authors declare no conflict of interest.

## ETHICS STATEMENT

Not applicable.

## Data Availability

Not applicable.
